# Query Matters: How Selection Strategies Influence Active Learning in Drug Discovery

**DOI:** 10.1021/acs.jcim.5c02504

**Published:** 2026-02-26

**Authors:** Huw J. Williams, Stephen D. Pickett, Andrew Baxter, David S. Palmer

**Affiliations:** † Department of Chemistry, 3527 University of Strathclyde, 295 Cathedral Street, Glasgow, G11XL, Scotland; ‡ GlaxoSmithKline R&D Pharmaceuticals, Stevenage, SG1 2NY, United Kingdom

## Abstract

We present SimDMTA, an *in silico* framework designed to simulate the Design–Make–Test–Analyze (DMTA) cycle used in preclinical drug discovery. Using docking scores as a proxy for biological assays, the simulations allow factors controlling the efficiency of the DMTA cycle to be explored in a manner that would not be feasible using traditional experiments due to time and cost constraints. In this workflow, a machine learning model predicts docking scores, selects compounds using various query strategies, docks selected molecules, and retrains iteratively. Starting from a broad chemical space, the model actively samples molecules derived from a 3,5-dimethyl-4-phenylisoxazole scaffold, an active warhead for the Bromodomain 4 (BRD4) BD1 binding site, to refine its predictions. Our results show that uncertainty-based sampling significantly outperforms greedy and hybrid approaches in both hit discovery and the ability of the model that predicts docking scores to generalize beyond its training set. Notably, by the final iteration, 37 of the top 50 ranked compounds were within the top 1% of the chemical space of all evaluated compounds. Strategies that include some random selection correct systematic biases more rapidly, but are less effective at predicting top-performing molecules. These findings underscore the value of incorporating molecular diversity and uncertainty into design strategies. While such strategies may deprioritize those molecules with the highest absolute predictions in early rounds, they markedly accelerate model refinement, ultimately leading to more effective hit identification in discovery driven by active learning.

## Graphical abstract

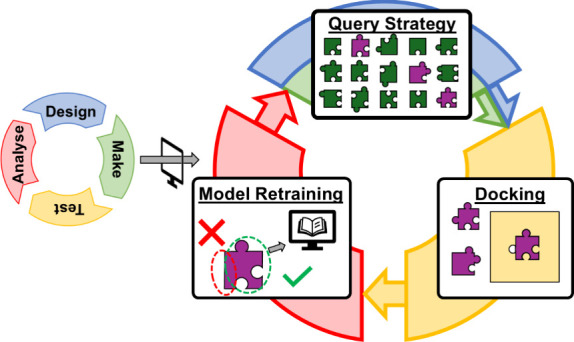

## Introduction

The Design–Make–Test–Analyze (DMTA) cycle is a widely adopted framework in preclinical drug discovery.^1,2^ It is used to systematically optimize chemical compounds to improve their efficacy, selectivity and pharmacokinetic properties as potential therapeutics. The process is iterative and consists of four key stages: designing molecules to engage a specific biomolecular target (*Design*), synthesizing the proposed compounds (*Make*), evaluating their pharmacological and physicochemical properties (*Test*), and using the resulting data to inform subsequent iterations (*Analyze*).

However, the DMTA cycle is inherently limited by both the time and the costs required to complete each iteration. The time taken for synthesis can vary significantly between molecules depending on factors such as the availability of starting materials, underlying synthetic precedent, or synthetic feasibility across a selected iteration. Typically, the synthesis of a new chemical entity (NCE) involves at least three reaction steps, but averaging around six.^3^ Assuming 1 day per step, a significant underestimate for chemistry requiring route scoping or condition optimization, this results in substantial turnaround times for each NCE. This timeline extends even further when molecules undergo assay testing. In-house testing at large pharmaceutical companies typically produces results within a week, while external testing can take much longer. Development of a new drug typically takes 10–15 years, with around four years spent in preclinical discovery, accounting for 30% of total development costs.^4^ The Research and Development (R&D) costs for a single drug molecule are between $160 million and $4.54 billion (2019 USD).^5^ With the extensive time and financial resources this framework requires, even small improvements in the processes may lead to large gains. However, it is challenging to optimize the DMTA cycle through experimental testing because of the number of factors that influence the outcome and the time and resources required to investigate each one.

Traditionally, medicinal chemists select molecules for synthesis based on predicted pharmacological properties such as binding affinity, ADMET (adsorption, distribution, metabolism, excretion, and toxicity) profiles, as well as synthetic feasibility.^6,7^ The primary goal is to identify promising drug candidates, but even compounds that do not advance can offer valuable insights for future design by providing a more complete characterization of the chemical space of greatest interest to a project. Today, Computer-Aided Drug Design (CADD) plays a central role in guiding compound selection. Artificial intelligence (AI), particularly machine learning (ML), is increasingly used to predict pharmacologically relevant properties such as target binding, toxicity, and pharmacokinetics.^8,9^

While ML models are becoming increasingly accurate, their performance remains heavily dependent on the quality and diversity of training data. However, the best compounds to synthesize, test and include in the training data may not always be the same ones that would traditionally be prioritized by medicinal chemists. Sampling based on predicted favorable traits such as potency or low toxicity is referred to as exploitative (or greedy) sampling. In contrast, explorative sampling involves selecting less characterized or structurally novel compounds to broaden chemical space coverage and improve the generalization of predictive models. Given the resource demands associated with synthesizing NCEs, it is unsurprising that discovery campaigns have historically favored analogues structurally similar to known actives. While this approach reduces the risk of costly synthesis of inactive compounds, it often results in imbalanced data sets and limits the ability to escape regions of chemical space where it is unlikely that a compound satisfying the full target profile will be found.

Balancing these two strategies is central to active learning (AL), a subset of ML in which an algorithm selects which data points to label from a pool of unlabeled candidates.^10^ This process is typically iterative, with models retrained as new data points are incorporated, iteratively refining the models. Candidates are chosen at each iteration using criteria collectively referred to as “query strategies”, “acquisition strategies”, or “selection methods”. In general, AL pipelines employ one of three approaches to sampling: purely exploitative, purely explorative, or a balanced strategy which integrates both.

AL is becoming increasingly valuable in pharmaceutical research, as its iterative structure complements the DMTA cycle. Consequently, there is a growing interest in optimizing these techniques for better integration into these pipelines. A key challenge in AL lies in the mismatch between the type of information gain valued by machine learning models and that required by medicinal chemists, namely predictive improvement versus structure–activity insight. The literature presents a wide range of query strategies, from simple greedy or uncertainty-based sampling to more sophisticated methods that estimate expected information gain, as well as hybrid approaches that balance multiple selection criteria.^11–15^ Given that each query strategy can have markedly different effects on the type and quality of information acquired, selecting an appropriate strategy requires careful alignment with the specific objectives of a given research campaign.^16^

Kangas et al. investigated various selection methods and demonstrated that balanced acquisition strategies offer the greatest promise for improved model performance.^17^ Here, they designed a hybrid strategy that balanced exploration and exploitation by alternating between the two methods at each iteration. They showed that sampling just 3% of the experimental chemical space was sufficient to recover 60% of the hits present in their data set. Similarly, Luo et al. found that using uncertainty sampling, which chooses molecules with high standard deviation across ensemble predictions outperformed purely greedy or random approaches.^18^ Unlike studies that use diverse data sets spanning multiple protein–ligand pairs and chemical scaffolds, the present work evaluates how active learning workflows evolve within a constrained chemical space, using a relatively small and specialized training set focused on a single protein and the same initial ligand scaffold.

In this study, we integrate a machine learning model and molecular docking within an AL framework to investigate how different query strategies influence knowledge gain across both computational models and the real-world DMTA cycle. By using molecular docking as a proxy for experimental IC_50_ assays, we eliminate the need for costly and time-consuming synthesis and laboratory testing. Although molecular docking scores are often not quantitatively correlated with empirical assay measurements, the resulting estimates and predicted molecular structures are physically grounded and interpretable, making them suitable as indicators of binding behavior.^19^ Using a Bromodomain 4 (BRD4) BD1 test system, we simulate the early stages of preclinical drug discovery, where data on new warhead-derived molecules are limited. We examine key considerations faced by medicinal chemists, such as how molecules are selected in these early stages, and how many molecules are needed to meaningfully improve predictive models, particularly when using those models to guide candidate selection. Our goal is to optimize molecule selection to enhance both computational and synthetic knowledge gain, ultimately improving the efficiency of the DMTA cycle in terms of cost, time, and decision-making. In this work, we evaluate a range of AL query strategies across an in silico DMTA workflow (SimDMTA) using a BRD4 BD1 test system. We evaluate the impact of these strategies on hit discovery, model performance, and bias correction, offering practical insights into how AL can support decision-making in real-world drug discovery, particularly when initiating projects with limited or nonrepresentative data.

## Experimental Section

### SimDMTA Workflow

SimDMTA is an algorithm that simulates the DMTA cycle within a computational framework. As no synthetic constraints are present, the *Design* and *Make* stages are combined into a single step. Two key factors were investigated: (1) the number of molecules selected per iteration, and (2) the query strategy used to guide selection and inform subsequent iterations. The complete SimDMTA workflow is shown in Figure 1, illustrating how the traditional DMTA cycle has been adapted for this study. To reduce sampling bias, every experimental result presented in the following sections is an average of three independent SimDMTA runs, each initiated with a different random seed; an analysis of the variability between repeated runs is provided in the Supporting Information under Figure S1.

**Figure 1:**
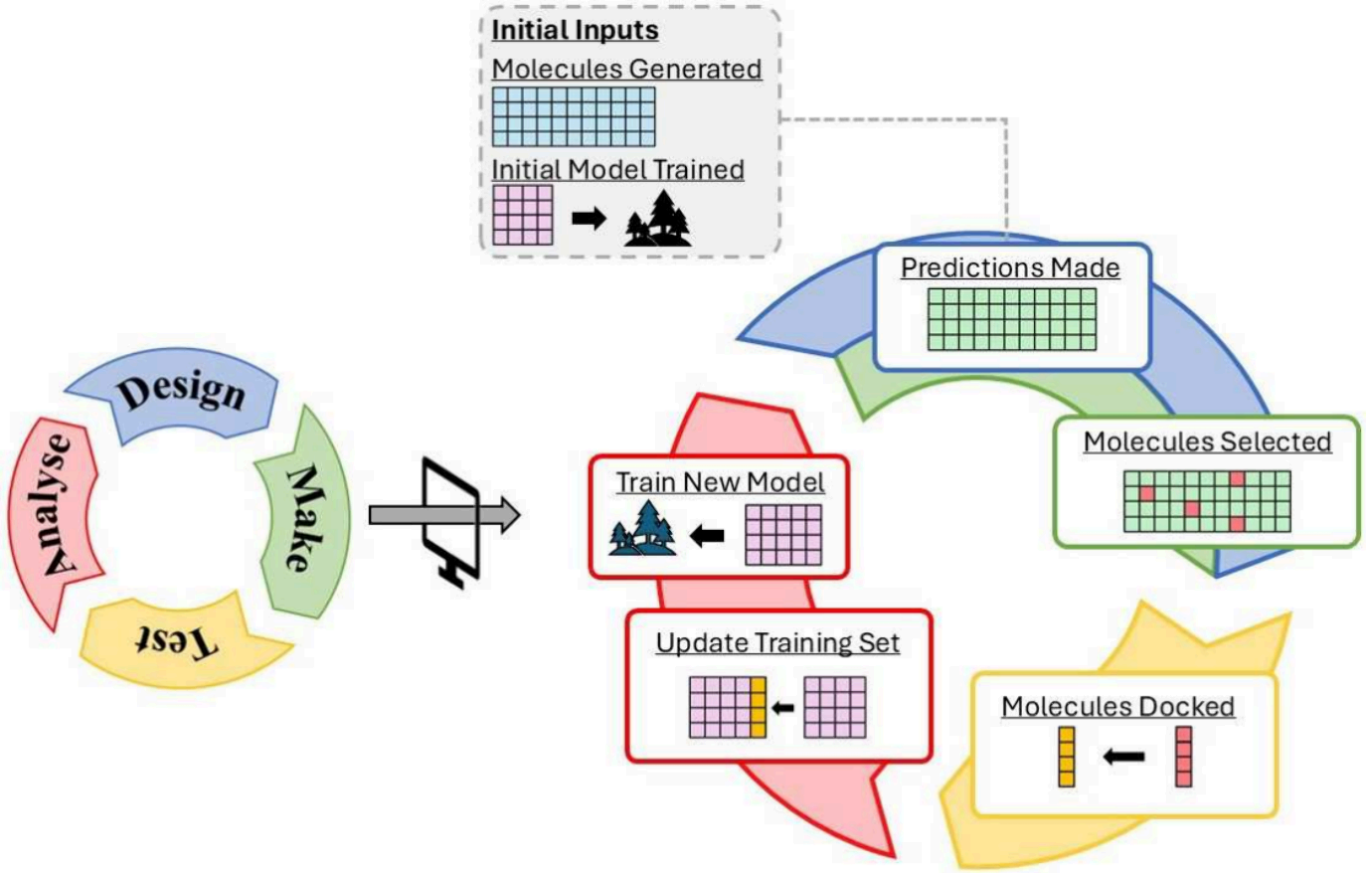
SimDMTA workflow illustrating the iterative nature of the algorithm.

### Data Collection

#### Training Data

The initial training data was curated from the ChEMBL database.^20,21^ Compounds were selected based on their activity against the BRD4-containing protein target with the ChEMBL ID: CHEMBL1163125. Molecules were first filtered based on the availability of IC_50_ values from human-derived binding assays prior to 2019, after which docking was performed to obtain the scores used for the initial training set. A comparison of the docking scores with the experimentally determined pIC50 values for all of the 1,054 molecules in the initial training set demonstrates that although absolute values differ, sufficient correlation exists to qualitatively differentiate between favorable and unfavorable binding behavior (Figure S2).

#### Molecule Selection Pool

The molecules in the selection pool were generated using some of the underlying methods in PyMolGen, a molecular generator developed by Falcone et al. PyMolGen constructs molecules by assembling ChEMBL-derived fragments onto predefined substitution points of a core scaffold. The core used in this study was a known BRD4-active moiety, 3,5-dimethyl-4-phenylisoxazole, shown in Figure 2. The fragment pool was created by systematically fragmenting molecules from the entire ChEMBL database.^20^ Molecules containing atoms other than C, N, O, S, H, F, Cl, Br, or I were excluded, along with ones with fragments known to interfere with bioassays, or too large to feasibly fit within the BRD4 BD1 binding site. Fragments containing more than three aliphatic rings were also taken out. In addition, disulfides, sulfones, and noncyclic sulfur-containing fragments were removed. The final fragment pool consisted of 16,310 unique fragments. Running the PyMolGen algorithm but restricting to parallel single fragment addition at two predefined attachment points resulted in a selection pool of 3,065,097 molecules after filtering as described in the next section.

**Figure 2:**
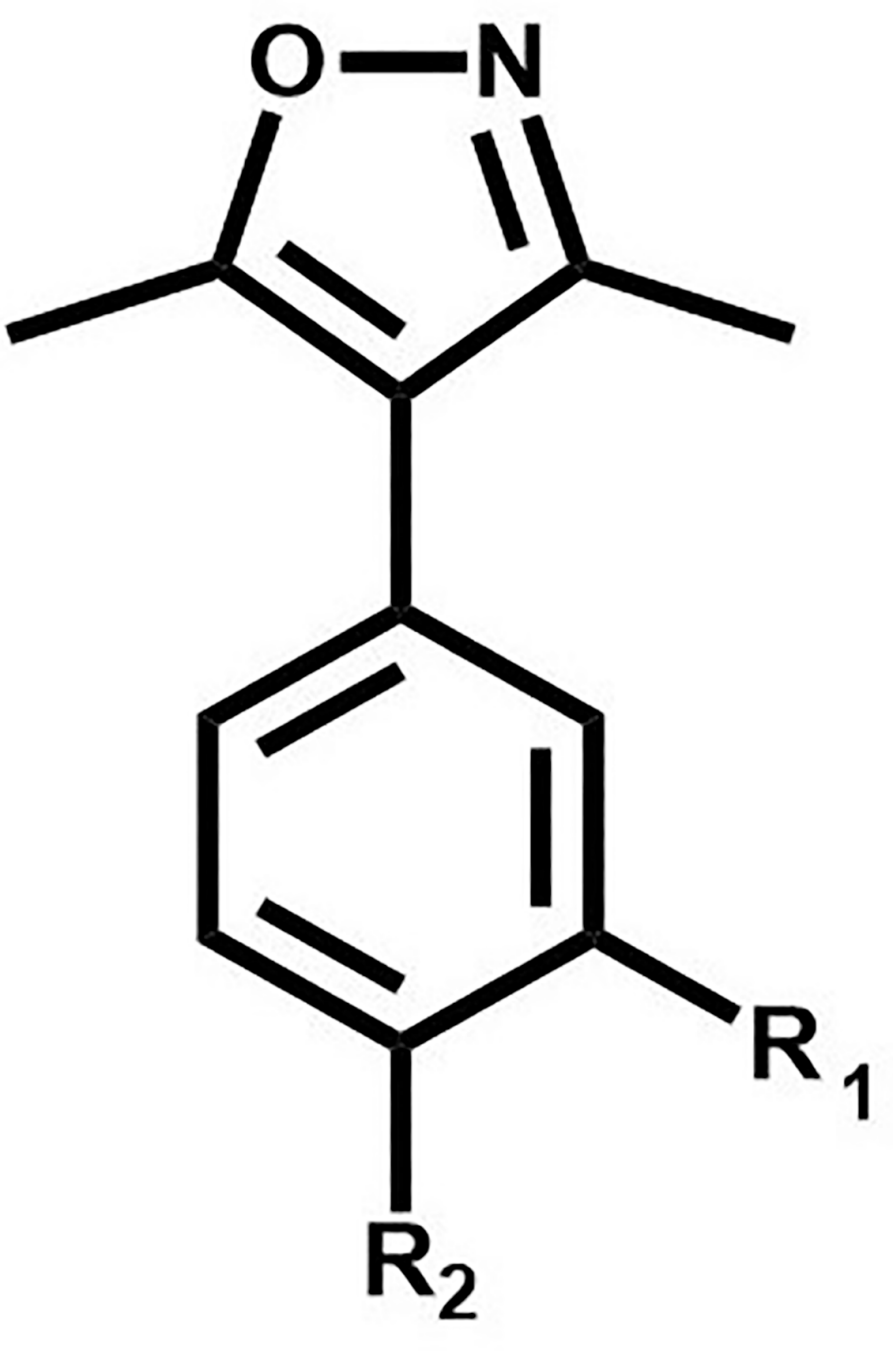
Substitution points at R_1_ and R_2_ on the 3,5-dimethyl-4-phenylisoxazole core (left) used during the molecule-building process to generate both mono- (R_1_ or R_2_) and di- (R_1_ and R_2_) substituted molecules.

#### Hold Out Test Sets

From the PyMolGen generated molecule set a randomly selected pool of 3000 molecules was docked to use as an external test set, of which 2945 were successful. From these, the molecules with the best 500 docking scores were used as a “top performing” test set.

### Data Preparation

#### Standardization

First, SMILES strings were enumerated through their tautomers and canonicalized using RDKit.^22^ The original active core was frozen to ensure the desired tautomer was maintained. Next, SMILES were adjusted to biological pH (7.4) using OpenEye and Kekulized with RDKit for compatibility with the Lilly-MedChem workflow.^23,24^ Once standardized, molecules were screened using the Lilly-MedChem rules and further filtered according to the criteria listed in Table 1, which aim to remove promiscuous compounds and those likely to fail in the drug discovery pipeline. Most of these filters are based on a relaxed version of Lipinski’s Rule of Five, designed to preserve chemical diversity.^25^ Of the 7.5 million molecules generated by PyMolGen, 3,065,097 remained after filtering, corresponding to a pass rate of 40.8%.

**Table 1:** Filters Used to Remove Molecules with Less Desirable Features

filter	passing condition
molecular weight budget (a.m.u)	≤600
No. of aromatic rings	≤3
PFI^c^	≤8
No. of hydrogen bond donors	≤6
No. of hydrogen bond acceptors	≤11

The Property Forecast Index (PFI), introduced by Young et al., quantifies how “fat and flat” a molecule is.^26^ It is calculated as the sum of the number of aromatic rings (NAR) and the chromatographic LogD. Prior studies have shown that drug candidates with PFI < 7 are more likely to meet multiple drug-likeness criteria. In this work, a modified PFI was computed using RDKit’s XLogP in place of experimental ChromLogD, allowing us to maintain a fully computational workflow. The modified PFI, denoted PFI^c^, is computed using eq 1.

$${\mathrm{PFI}}^{c}=\log_{10}P+N_{\mathrm{aromatic rings}}$$ 1

### Descriptor Calculation and Molecular Representation

Molecular descriptors were generated using RDKit. A total of 210 2D descriptors were calculated for each molecule using their SMILES representation.^22^ These descriptors included physicochemical, topological, constitutional, electronic, and geometrical properties, as well as fragment-based descriptors that quantify the presence or frequency of specific molecular substructures (e.g., functional groups, ring types, or atom environments).

To represent the 3D coordinates of molecules for docking, structural data files (SDFs) were generated. This was done by first explicitly adding hydrogen atoms to each molecule and converting their structure into an SDF-compatible MolBlock format using RDKit’s MolToMolBlock.^22^ The ionization state was then adjusted to reflect the predominant form at biological pHs using OpenEye’s p*K*_a_ modeling tools.^23^

### Protein–Ligand Docking

The BRD4 BD1 biological target was obtained from the RCSB Protein Data Bank (PDB ID: 4BW1).^27^ The binding site was defined using GNINA’s auto box finder on the original 4BW1 complex, which contains a 3,5-dimethylisoxazole ligand.^28^ The coordinates in angstroms of the center of the binding site were (*x*, *y*, *z*) = (14.66, 3.41, 10.47). Ligands and water molecules were removed, except for those involved in the binding modality (residue numbers A2086, A2087, A2106, A2139, A2117). The receptor was prepared using Open Babel (v3.1.1). Protonation states were determined based on physiological pH, followed by the addition of hydrogen atoms and assignment of partial atomic charges. The resulting structure was then converted to the GNINA-compatible PDBQT format. Docking scores were calculated using GNINA 1.0, with the precompiled binary sourced from its official GitHub repository (https://github.com/gnina/gnina).^29,30^ The docking box was centered on the BRD4 BD1 binding site with dimensions (17.67 × 17.00 × 13.67 Å). To reduce computational cost, flexibility was limited only to side chains within the binding site. Docking exhaustiveness was set to 8, allowing up to 8 pose search tasks to be run, and the number of conformers per task was limited to 9, equating to a maximum of 72 poses per molecule. Each pose was generated by perturbing torsion angles, making small translations and rotations, and then evaluating the docking score. The docking score (reported as binding affinity in kcal/mol) was used as the pseudoexperimental value, which is calculated using the AutoDock Vina scoring function.^31^

Across the full experiment, a total of 147,248 molecules from the PyMolGen library were subjected to molecular docking, of which 97,289 produced valid docking scores.

### Machine Learning Models

Random Forest (RF) models were built using the RandomForestRegressor class from scikit-learn (version 1.5.1).^32^ The initial model was trained on RDKit descriptors derived from 1,054 ChEMBL BRD4 inhibitors to predict the docking score of candidates in kcal/mol. Model optimization was performed via nested cross-validation, with a 5-fold inner loop for hyperparameter tuning using a grid search, and a Monte Carlo outer loop with 50 resamples of a 70:30 train-test split to assess performance.

The RF hyperparameters that were optimized during each inner loop were: the number of estimators (400 or 500); maximum tree depth (10, 20, 30, or 50); and the minimum samples required to split a node to form a leaf (10, 20, or 30). The number of features considered in each split was set to $\sqrt{\mathrm{number of features}}$ , promoting diversity among trees. No restrictions were imposed on the number of leaf nodes and no additional weighting was applied. No pruning techniques were used.

### Model Evaluation

#### Model Performance Metrics

During training, hyperparameters were optimized to minimize the mean squared error (MSE), evaluated using 5-fold cross-validation (CV) on the training set. The predictive accuracy of the trained models was quantified using four evaluation metrics, namely root mean squared error (RMSE), bias, standard deviation of the error of prediction (SDEP), and the Pearson R. The equations for these metrics and MSE can be found in the Supporting Information under eqs 1–5.

#### Hit Evaluation

To evaluate the effectiveness of each query strategy in identifying potent candidates, we defined a hit as any molecule within the top 1% of docked compounds (*n* = 973), based on actual docking scores. At every iteration, the 50 molecules with the best predicted docking scores (as ranked by each model) were selected and compared against the predefined hit set. The number of overlapping molecules was used to assess each strategy’s ability to prioritize potent compounds.

### Query Strategies

Several experiments were conducted to investigate the impact of different molecule selection strategies on model performance. Additionally, two selection batch sizes were evaluated: 10 and 50 molecules per iteration. The selection methods and their corresponding abbreviations are listed in Table 2.

**Table 2:** Default Query Strategies and Their Abbreviations

selection method	abbreviation
random	R
best predicted docking score	MP
best predicted MPO	MPO
highest prediction uncertainty	MU
random selection in top 10% best predicted docking scores	RMP
random selection in top 10% best predicted MPO	RMPO
random selection in top 10% most uncertain	RMU
hybrid selection using both MP and MU	MP:MU
hybrid selection using both RMP and RMU	RMP:RMU

The multiparameter optimization (MPO) value derives from the following equation, penalizing molecules that have a less drug-like character.

$$\mathrm{MPO}=\frac{-P_{m}}{1+e^{{\mathrm{PFI}}_{m}^{c}-8}}$$ 2

where *P*_*m*_ is the predicted docking score of molecule, *m*, and PFI_*m*_^c^ is the PFI^c^ of *m*. The MP and MPO strategies are analogous to previously described “greedy” approaches, while MU represents an explorative search. Although RMP and RMPO incorporate both randomness and exploitation, they are best characterized as guided-exploration strategies. This approach was designed as a more effective alternative to purely random selection. By sampling randomly within more promising regions of chemical space, the AL process is still theoretically driven toward more desirable molecular candidates, thus remaining aligned with the objectives of real-world DMTA cycles.

The prediction uncertainty, σ, for a given molecule is calculated as the standard deviation across all decision trees in the RF:

3 $$\sigma =\sqrt{\frac{1}{T}\overset{T}{\underset{t=1}{\sum }}{\left({ŷ}_{t}-{ŷ}_{\mathrm{mean}}\right)}^{2}}$$

where *T* is the number of trees in the RF, *ŷ*_*t*_ is the predicted value of the *t*-th tree, *ŷ*_mean_ is the average prediction across all trees.^33^ This definition of uncertainty has been used in similar work and has shown to have similar performances to other uncertainty functions such as entropy sampling, expected information gain (EPIG), and latent space or feature space distances.^34,35^

Next, we investigate hybrid query strategies that combine two individual selection methods within the same iteration. While previous studies typically alternate strategies across standalone iterations, our approach selects molecules using both strategies concurrently, according to predefined contribution ratios.^12,13^ Specifically, we constructed two hybrid strategies: MP:MU and RMP:RMU. To assess how these combinations influence model performance, we evaluated each hybrid under three contribution ratios: 8:2, 5:5, and 2:8. For example, in the MP:MU hybrid, an 8:2 ratio means that out of 10 molecules selected per iteration, 8 are chosen based on predicted potency (MP) and 2 based on predictive uncertainty (MU). This setup enables us to explore the effect of varying the contributions of each component strategy on the overall learning process.

Finally, we evaluated the impact of varying the selection pool size within the RMP strategy, assessing five thresholds: 2.5%, 5%, 10%, 25%, and 50%. Each threshold corresponds to the proportion of PyMolGen-generated molecules available for selection after ranking by their respective acquisition values.

### Computation vs Synthetic Turnaround Times

All experiments were conducted on a system with 40 CPU cores. During the docking phase, the algorithm submitted one job per molecule, with each job using a single core. Once all docking jobs were completed, the algorithm proceeded to the next portion of the cycle, model training. Parallel docking was performed in addition to the 40-core main workflow. On average, a single iteration selecting 10 molecules required approximately 2,250 s (∼37.5 min) to complete. Extrapolating this to a full set of 150 iterations resulted in an average total runtime of 93 h 45 min. For experiments that selected 50 molecules, the runtime per iteration increased to 2466 s (41.1 min). However, because only 30 iterations were required to add an equivalent number of molecules to the training set, the overall time was reduced to an average of 20 h 33 min.

Considering a real-life DMTA cycle with an average of six reaction steps with each taking 1 day, and a seven-day turnaround for *in vitro* examination, the minimum time required for a single cluster of novel, structurally similar molecules is 13 days. Assuming 10 molecules were synthesized within this time frame, completing 150 iterations would require 1,950 days of runtime which is the equivalent of 15,600 working hours. This underlines the importance of effective molecule selection for early drug discovery, and solidifies the justification of integrating CADD into these processes. However, integration of these tools requires refinement to better align with the capabilities and focuses of synthetic stages. Our focus here is to assess the extent to which ML model improvement is influenced by the empirical outputs of DMTA iterations, and to understand how selection strategies can be designed to complement holistic project learning versus chasing short-term improvements. We aim to suggest alternative approaches to selecting the molecules to synthesize. Ultimately, our goal is to help shorten drug discovery time frames, reduce costs, and accelerate the availability of new treatments.

### Data Set Analysis

The PyMolGen-generated structure pool exhibits a broader range of features compared to the ChEMBL and held-out test sets. This is reflected in the principal component analysis (PCA) of the 210 2D molecular descriptors, as shown in Figure 3. This is to be expected considering that the ChEMBL training set consists of only known BRD4 inhibitors, a fraction of the entire ChEMBL database. Additionally, the held-out test set is only 0.1% of the entire PyMolGen set. The combinatorial approach used in PyMolGen results in the molecules generated having a wider distribution of features. Some regions of the feature space occupied by the ChEMBL molecules remain unrepresented by the other PyMolGen sets. This derives from the filters that are applied after molecule generation to remove 3- and 4-membered rings and limit the number of fused/aromatic rings. Given that the 3,4-dimethyl-4-phenylisoxazole seed has two aromatic rings, the chemical diversity achievable within these constraints is limited. The most populated feature space across all three data sets has similar distributions, as seen in the kernel density estimate (KDE) plots on the diagonal of Figure 3.

**Figure 3:**
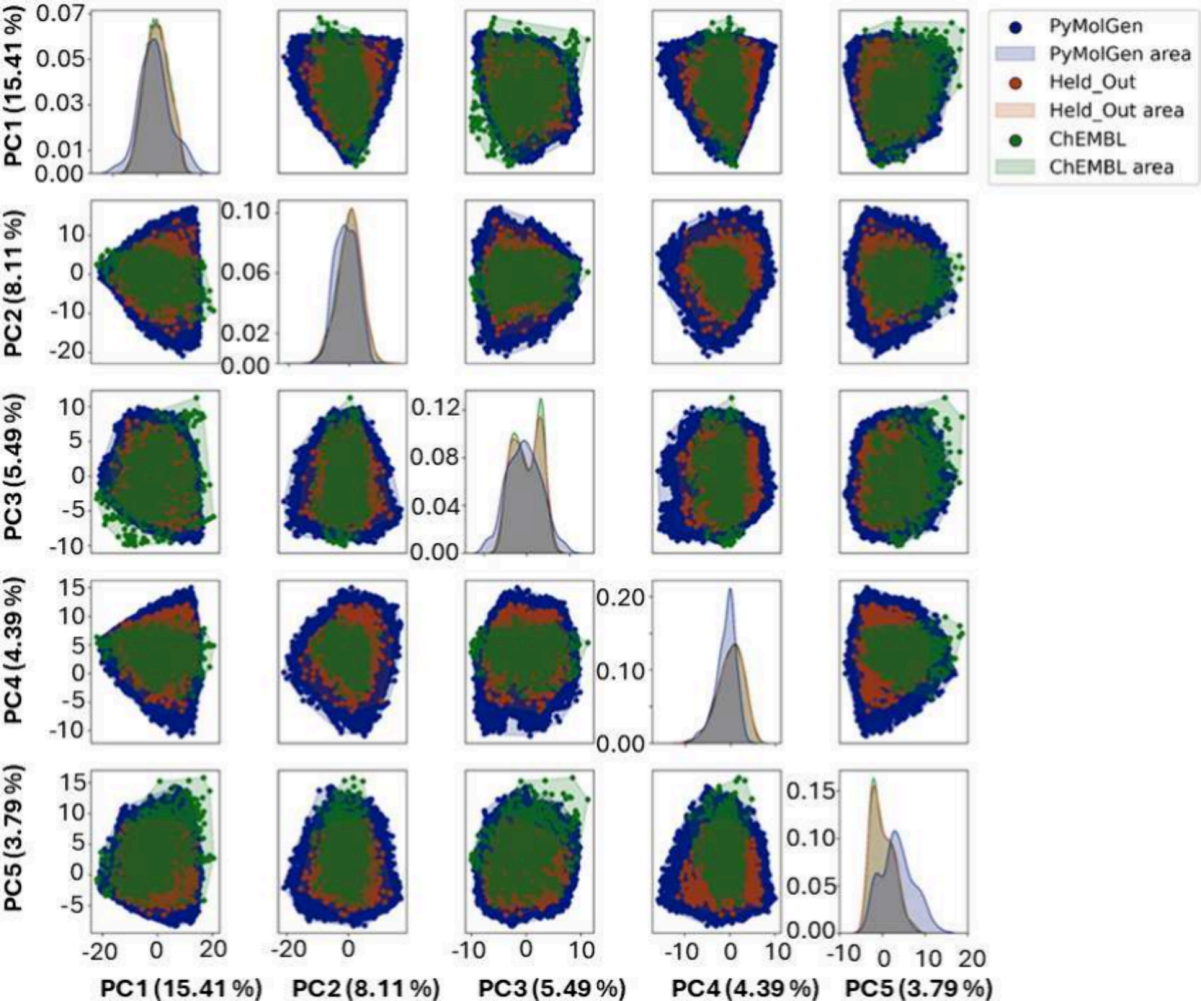
Principal component analysis (PCA) plot matrix for the three data sets: PyMolGen (blue), Held-Out Test Set (orange), and ChEMBL (green). Kernel density estimate plots are shown along the diagonal. PCA was performed using the 210 2D RDKit molecular descriptors calculated for each data set.

## Results and Discussion

### Effect of Selection Strategy on Hit Discovery Potential

Greedy acquisition strategies (MP and MPO) result in models with limited ability to identify new hits. In contrast, uncertainty-based sampling (MU) demonstrates an excellent capacity for discovering actives, as shown in Figure 4a. This strategy ultimately achieves a hit rate of 74% within the top 50 predicted molecules after 1,500 additions to the training set, indicating that more than two-thirds of selected compounds are true high-affinity binders. The hit rate here is defined as the proportion of selected compounds that fall within the predefined hit set. Greedy methods do not reach comparable levels of enrichment, underscoring the advantage of exploration-driven sampling in early stage discovery. Although all random-incorporating strategies result in comparable hit rates at the end of the experiment, the hit rate accumulation trajectory of RMU is notably slower than the other strategies, as seen in Figure 4b. Interestingly, purely random selection outperformed top 10%-constrained random sampling, highlighting that rigid ranking can limit chemical diversity even within exploration-based methods. This suggests a more nuanced interplay between exploration and exploitation than traditionally assumed.

**Figure 4:**
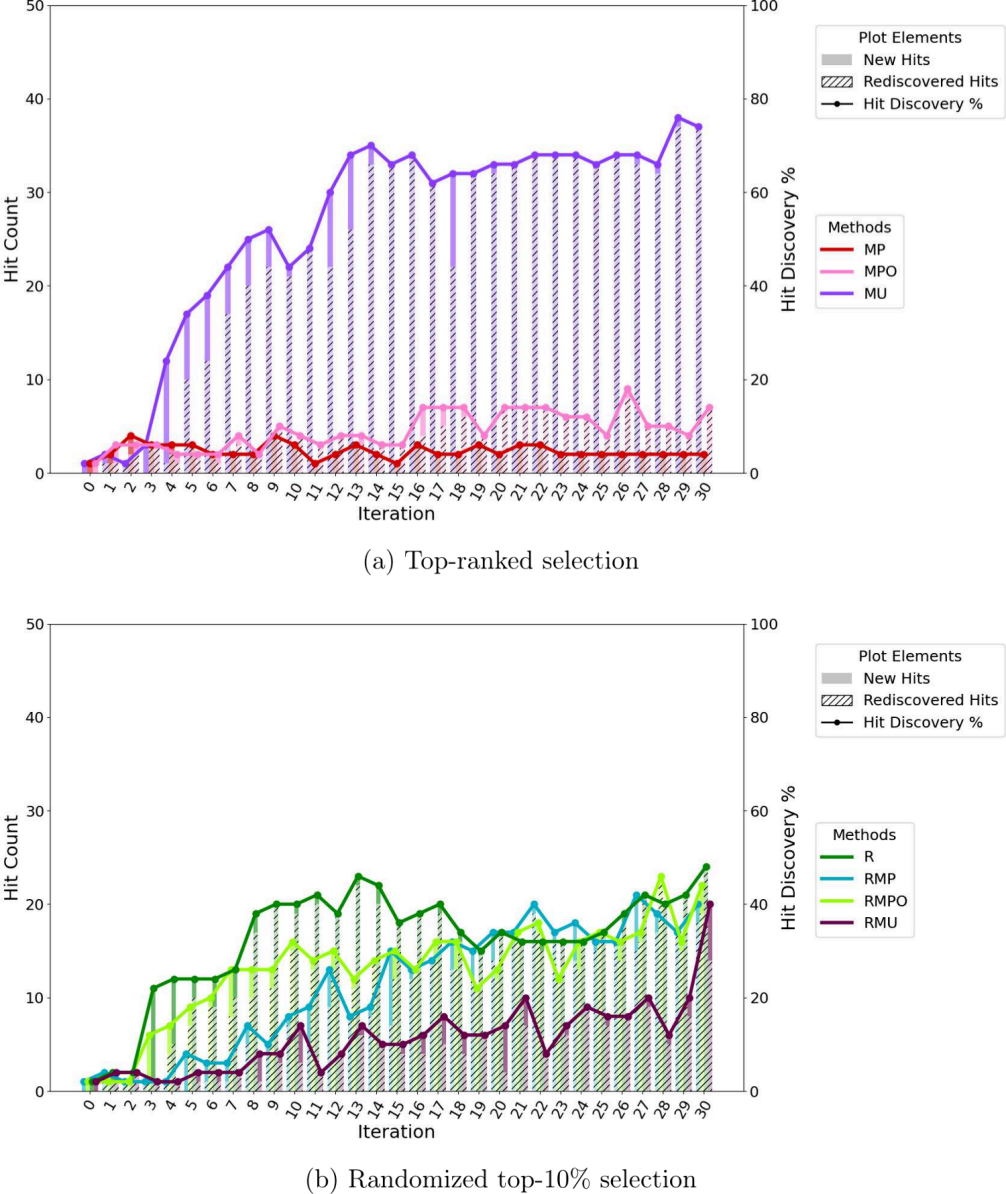
Hit discovery performance of acquisition strategies across iterations (batch size = 50). Hits are defined as the top 1% of all docked compounds based on actual docking scores. (a) Compounds selected strictly by top acquisition scores (MP, MPO, MU). (b) Compounds selected either purely at random or randomly within the top 10% of each acquisition score ranking.

Although MU sampling consistently achieved higher overall hit discovery rates, the RMP and RMPO strategies occasionally identified the single best-scoring compounds, showing the most favorable individual docking scores (see Figures S2–S4 in the Supporting Information). However, these cases were rare, showing that only one or two molecules per experiment exceeded the docking scores of those selected by the MU.

Analysis of the synthetic accessibility (SA) was carried out using the SAScore proposed by Ertl et al. (Supporting Information, Figure S5).^36^ The generated compound set exhibited minimum, maximum and mean SA scores of 1.72, 6.49, and 3.25, respectively, with the 3,5-dimethyl-4-phenylisoxazole scaffold scoring 1.74. Greedy selection methods generally queried molecules that were more synthetically accessible than those selected via MU; however, the difference was minor, with only a 0.6-unit gap. Although uncertainty-based methods select molecules with higher SA scores than the overall mean, these scores still fall within the “moderately easy to synthesize” range (2–4). It is possible that, in a broader chemical space not preseeded by a highly synthesizable moiety, this disparity of SA scores between these selection methods may become more pronounced.

### Effect of Selection Strategy on Model Performance

Models trained solely on exploitative acquisition strategies, such as MP and MPO, show strong internal performance but limited generalization at each iteration of the simulated DMTA cycle. As shown in Figure 5a, their internal RMSE decreases as more iterations of the DMTA cycle are carried out with the selection of molecules with high predicted docking scores. This results in the reinforcement of already well-characterized regions of chemical space and limits structural diversity.

**Figure 5:**
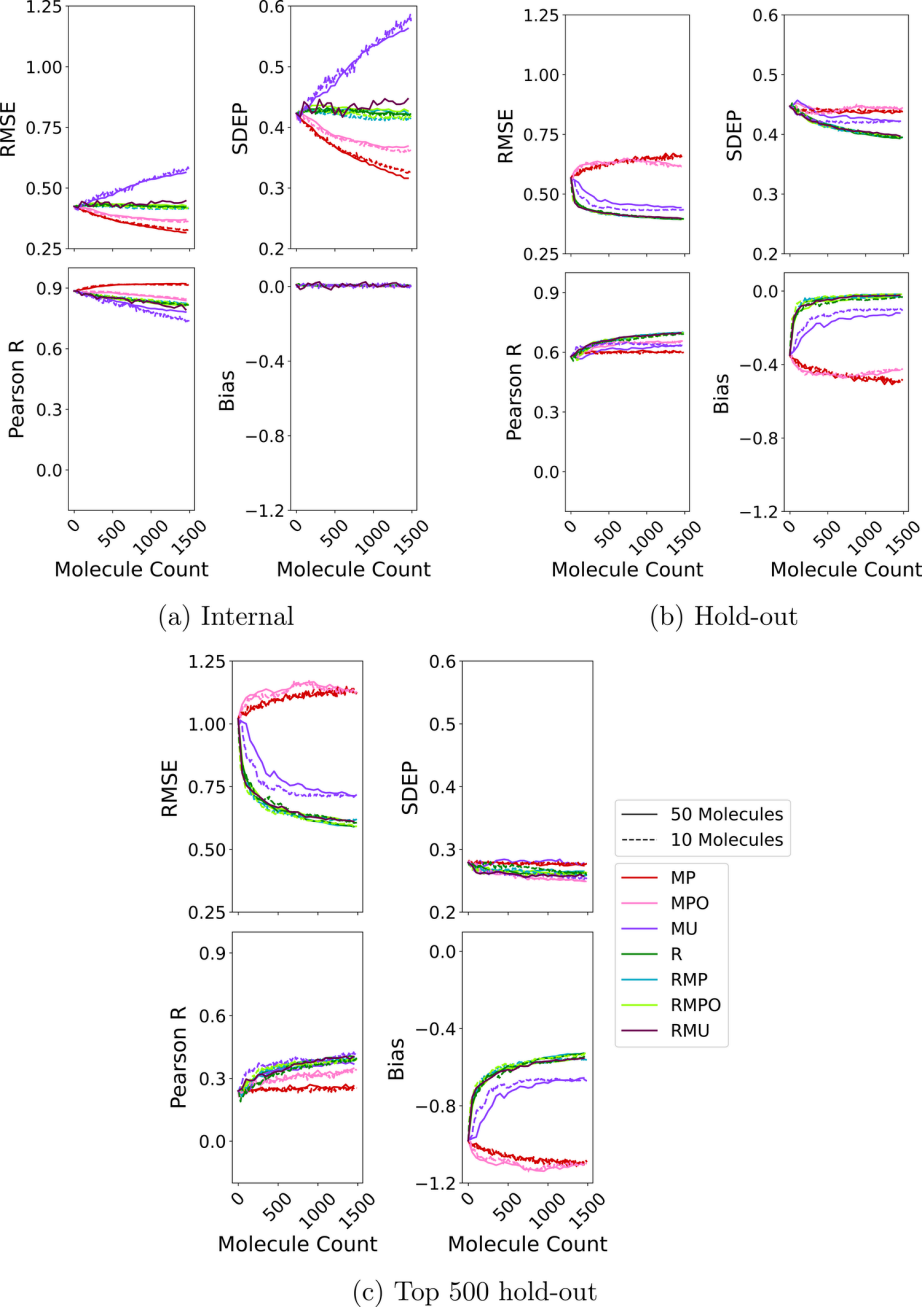
Average predictive performance across all query strategies: (a) internal retraining performance; (b) performance on the full hold-out test set; (c) performance on the top 500 molecules with the highest true docking scores. Internal metrics are averaged across MCCV folds. The hold-out set consists of randomly selected molecules excluded from the acquisition pool. The *x*-axis shows the cumulative number of molecules added to the training data during active learning.

These models perform poorly on the randomly selected hold-out test set, where RMSE increases with additional training data (Figure 5b). This trend indicates a failure to overcome the structural and property biases inherited from the ChEMBL-derived training set. To assess predictive accuracy on compounds of highest relevance to drug discovery, we evaluated model performance on 500 compounds from within the held-out set which had the best docking scores. The trends mirror those seen across the full test set but with more pronounced differences between acquisition strategies. The initial RMSE on the focused subset is around double that of the full hold-out set (1.02 kcal/mol vs 0.57 kcal/mol) and it increases by ∼0.1 kcal/mol over the course of the DMTA cycle, as can be seen in Figure 5c. This emphasizes the inability of exploitative models to generalize to high-affinity compounds.

This behavior is further reflected in learned feature distributions, which narrow over time (Figure S6, Supporting Information), demonstrating overfitting to a limited chemical subspace. In contrast, models trained with uncertainty-based strategies maintain broader feature exploration (Figure S7, Supporting Information), facilitating exposure to more diverse chemotypes. Moreover, the pairwise Tanimoto similarity among molecules in the training set provides additional evidence. Greedy selection strategies yield higher molecular similarity in selected compounds compared to explorative methods such as MU and R (Figure S8, Supporting Information).

The predictive performance of models using random-incorporating strategies (i.e., R, RMP, RMPO and RMU) is also notably consistent across all three test sets (internal, hold-out, and top-500 hold-out), suggesting that the inclusion of stochasticity helps maintain generalization even as the training data grows.

### Does Training the ML Model on CHEMBL Data Rather than Random Data Improve the Outcome?

To assess the potential influence of initial model bias, each query strategy was also evaluated using a control model, specifically, a random forest trained on the ChEMBL data set with randomly shuffled input features. This design disrupted the feature–target relationship, allowing us to examine whether the performance gains observed were influenced by biases inherent to the initial training data.

Unsurprisingly, the disruption had a pronounced effect on hit discovery. As shown in the Supporting Information (Figures S9 and S10), removing the feature–target correlation substantially impaired the model’s ability to identify compounds with favorable docking scores across all iterations of the simulated DMTA cycle. This indicates that useful information was indeed present in the original ChEMBL data set, which contributed to improved hit selection.

Under these scrambled conditions, greedy acquisition strategies (MP and MPO) failed to identify any hits over 30 simulated iterations. In contrast, the explorative MU strategy retained some discovery capability, achieving 40% hit identification within the top 50 predicted compounds by the final iteration (Figure S9, Supporting Information).

Interestingly, despite the lack of meaningful features, the overall predictive performance trends (RMSE and Pearson correlation) remained broadly consistent with those of the nonscrambled models. Final RMSE values for exploitative strategies were slightly improved (e.g., MP: 0.60 vs 0.65 kcal/mol; MPO: 0.55 vs 0.62 kcal/mol), though these differences may not be meaningful in practice.

These results suggest that while initial model bias had a substantial impact on hit discovery, it did not significantly affect the predictive performance trajectories of the acquisition strategies. Complete results are provided in the Supporting Information (Figures S10 and S11).

### Does Sample Size Matter?

There is a divide in the literature regarding whether the sample size chosen at each AL iteration significantly impacts the accuracy of the model and hence the hit-discovery performance.^13,37–39^ In our single-query strategy experiments, we observed no notable performance difference between batch sizes of 10 and 50. During exploitative sampling batch sizes of 50 are more likely to include more molecules with similar feature distributions due to the model’s prediction behavior. However, this behavior has been shown to have minimal impact on the overall predictive performance. Since both fall within the commonly recommended range, and using 50 is more computationally efficient, we chose to proceed with a batch size of 50 for the analyses presented here.

### Can We Balance Explore and Exploit?

In experiments where varying ratios of exploration and exploitation were combined within the same iteration, hybrid strategies were tested. Their hit-finding performance was comparable to that of purely greedy sampling across all selection ratios, as shown in Figure 6. The MU method continues to show superior hit discovery rates over these methods.

**Figure 6:**
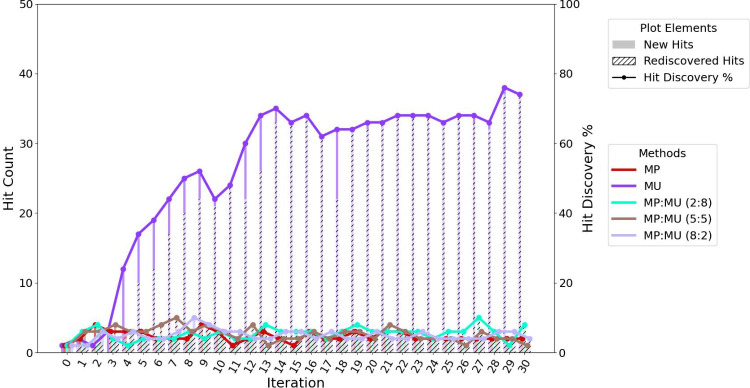
Hit discovery performance of the MP:MU hybrid acquisition strategy (batch size = 50), compared with individual MP and MU strategies. Hybrid strategies were evaluated at three selection ratios: 2:8, 5:5, and 8:2 (MP:MU).

This raises the question of why incorporating exploration alongside exploitation does not lead to improvement over solely exploitation-based methods, given that explorative methods were earlier indicated to be more performant. To investigate, we analyzed the predictive uncertainty throughout the PyMolGen set, grouped into prediction bins: −9.86 to −8.87 kcal/mol, −8.87 to −7.88 kcal/mol and −7.88 to −6.89 kcal/mol. We found that the lowest predicted docking score bin (i.e., the most favorable) consistently exhibited the highest mean uncertainty across all iterations, as shown in Figure 7. In this bin, the MP selection strategy led to rapidly increasing model uncertainty over DMTA iterations, whereas introducing a hybrid MP:MU selection strategy led to a slower increase in uncertainty. By contrast, using the MU selection rule decreased the model uncertainty. MP and MP:MU selection strategies led to less diverse feature sets than the MU strategy. This is evidenced by the evolution of feature distributions across strategies, shown in Figures S12–S14 (Supporting Information). For example, when we examine the fourth-order connectivity index (Chi4n), a topological molecular descriptor that quantifies branching and connectivity, we see that the hybrid strategies exhibit greater diversity in Chi4n compared to MP alone, but their distributions remain more similar to MP than to MU. As with MP, this results in reduced feature diversity within the hit space, contributing to poorer hit-finding performance. This overlap in sampling space between hybrid and greedy query strategies also accounts for their similar model accuracy, as shown in Figure 8. The full set of performance curves for the hybrid strategies is provided in Figures S15–S17 (Supporting Information).

**Figure 7:**
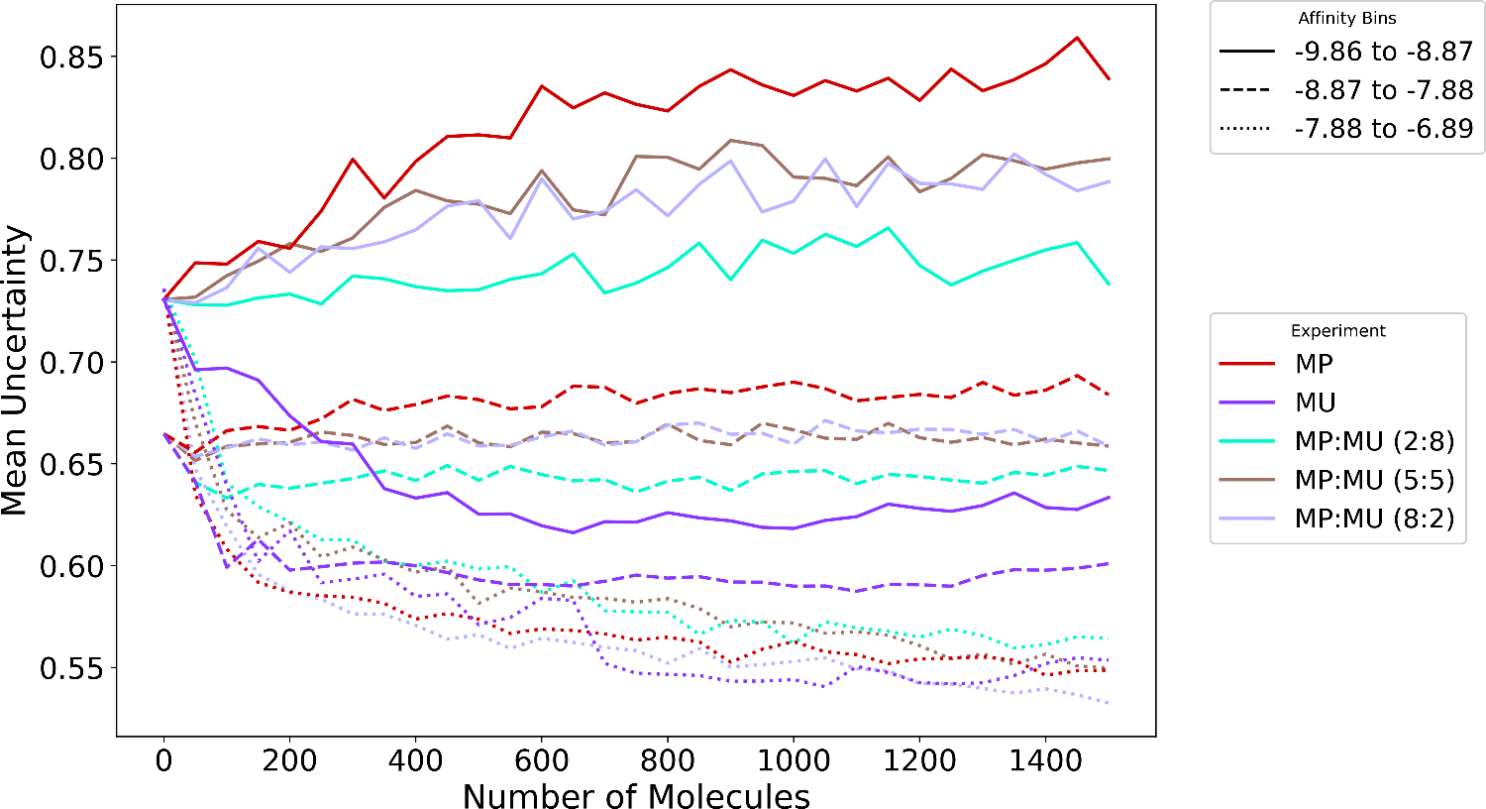
Evolution of mean prediction uncertainty over 30 iterations. Predictions are grouped into bins to highlight differences across prediction pools, with bin thresholds defined as −9.86 < *x* ≤ −8.87 kcal/mol, −8.87 < *x* ≤ −7.88 kcal/mol, and −7.88 < *x* ≤ −6.89 kcal/mol. The *x*-axis shows the cumulative number of molecules added to the training data during active learning.

**Figure 8:**
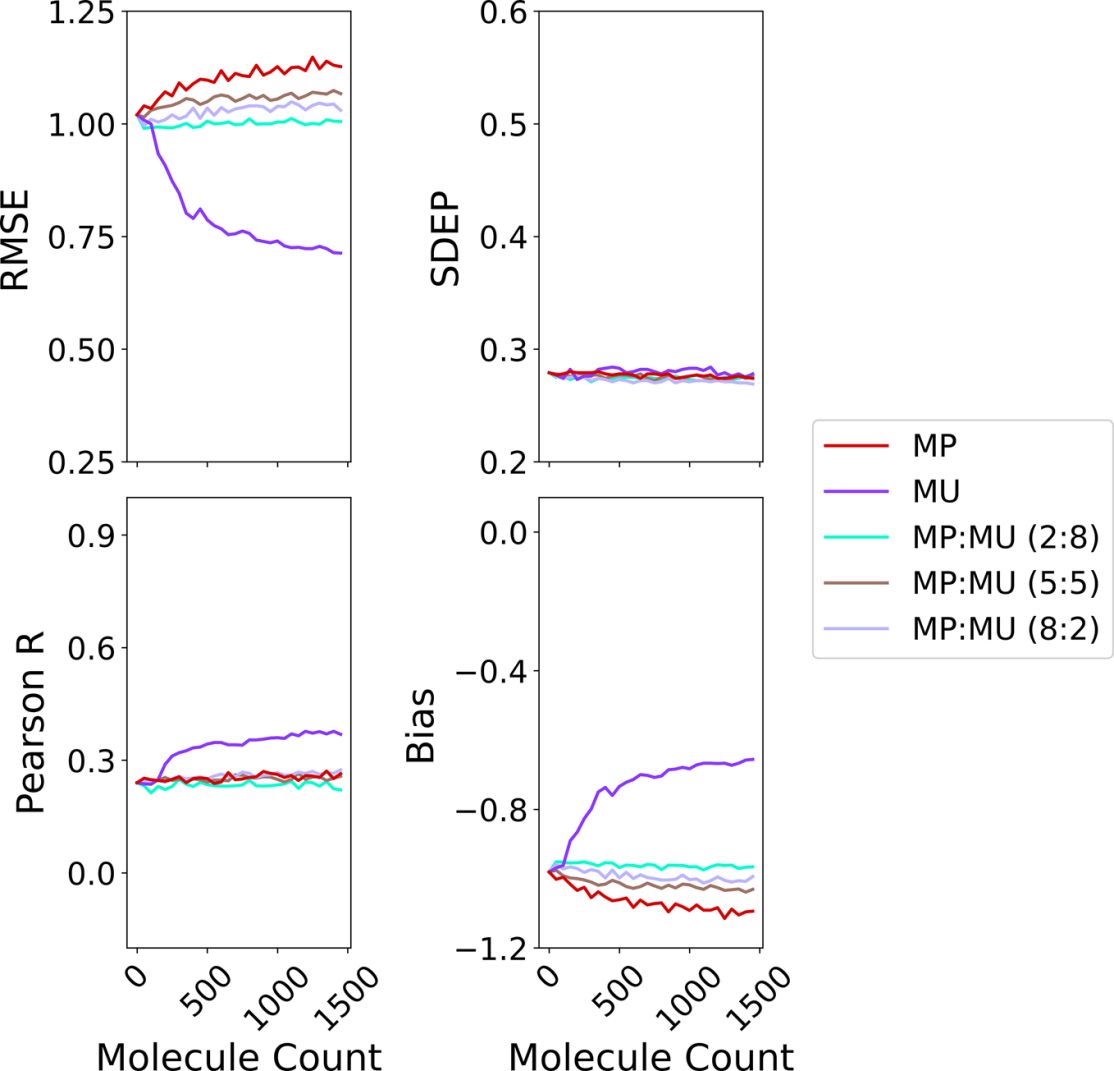
Average predictive performance across all query strategies (batch size = 50), evaluated on the top 500 molecules with the best true docking scores from the hold-out test set. The *x*-axis shows the cumulative number of molecules added to the training data during active learning.

When evaluating the hybrid strategy that combines RMP and RMU, we observed no significant difference in predictive accuracy compared to single pronged random-incorporating strategies such as R, RMP, RMPO, or RMU as shown in Figure 9. In terms of hit discovery, the addition of greedy-guided exploration resulted in modest improvements over purely random sampling. However, there was no consistent evidence that these improvements correlated with the selection ratios used.

**Figure 9:**
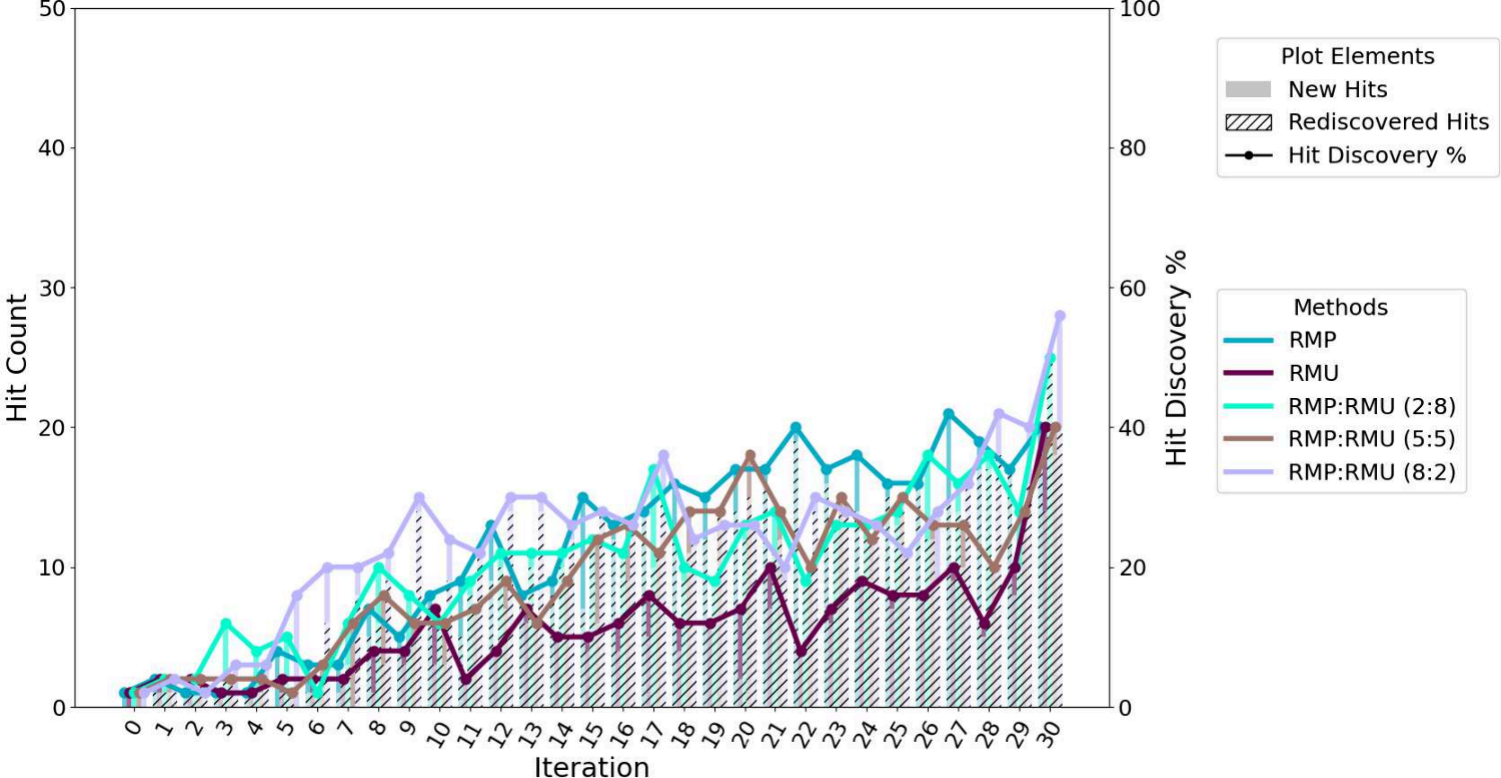
Hit discovery performance of the RMP:RMU hybrid acquisition strategy (batch size = 50), compared with individual RMP and RMU strategies. Hybrid strategies were evaluated at three selection ratios: 2:8, 5:5, and 8:2 (RMP:RMU).

### Does Selection Pool Size Matter?

To better understand the behavior of guided-exploration methods (RMP and RMPO), we analyzed their performance under varying selection pool sizes. Specifically, we evaluated seven pool sizes: the absolute top 50 molecules (equivalent to MP), and the top 2.5%, 5%, 10% (default for RMP), 25%, 50%, and 100% (equivalent to random sampling, R) of the ranked compound list.

We found that as the selection pool size decreased, model behavior increasingly resembled that of purely greedy sampling. However, this trend was not strictly linear. For example, the performance gap between the 2.5% and 5% pool sizes, shown in Figure 10, suggests a sharp shift in behavior. This indicates that compounds within the top 2.5% of predicted docking scores likely share highly similar feature profiles, which reinforces model bias and limits exploration.

**Figure 10:**
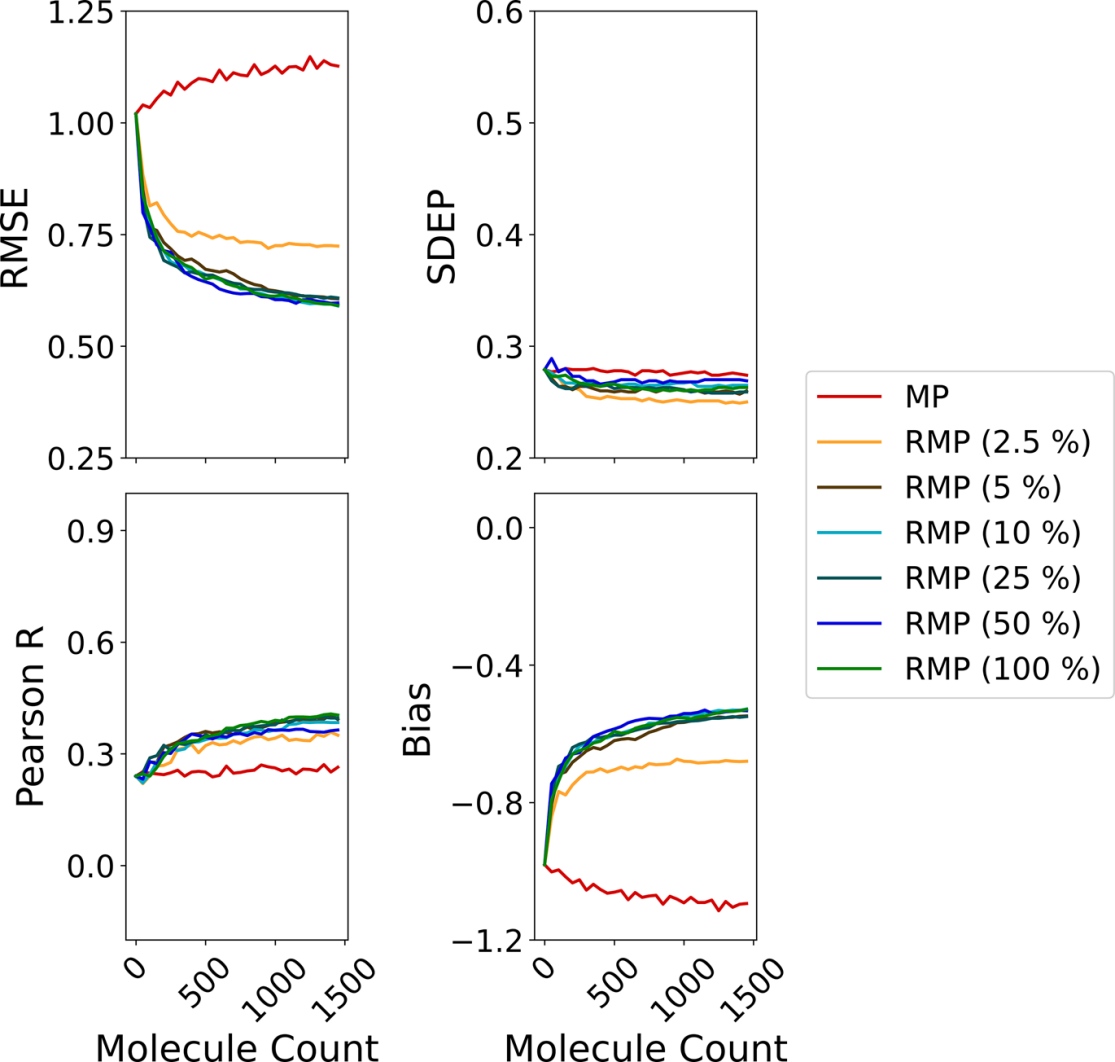
Average predictive performance of the RMP query strategy (batch size = 50) across varying selection pool sizes: 2.5%, 5%, 10% (default), 25%, 50%, and 100% (random, R). Performance was evaluated on the top 500 compounds with the best true docking scores from the hold-out test set.

Although the performance trajectory for the 2.5% pool is similar in shape to that of the MU strategy, its ability to discover hits is much lower, as shown in Figure 11. While some pool sizes appear to perform better than others, the high level of inherent randomness in these experiments makes it difficult to draw firm conclusions about which pool size is truly optimal.

**Figure 11:**
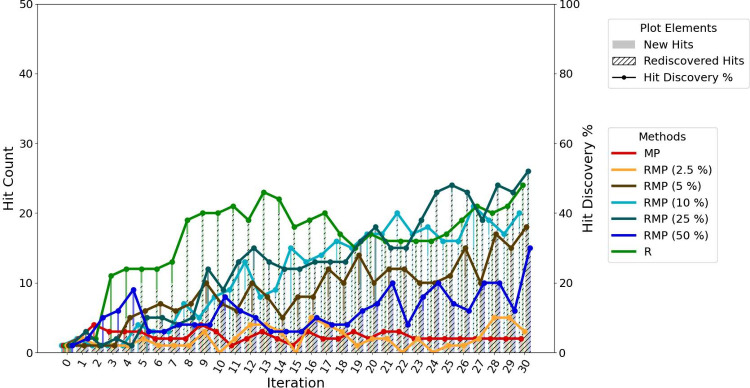
Hit discovery performance of the RMP query strategy across varying selection pool sizes (batch size = 50): absolute top (MP), 2.5%, 5%, 10% (default), 25%, 50%, and 100% (random, R). Performance was evaluated on the top 500 compounds with the best true docking scores from the hold-out test set.

### What is the Relationship between Error and Uncertainty?

While uncertainty-based sampling has shown strong performance in hit discovery, it is essential to examine whether predicted uncertainty reliably correlates with actual prediction error. When evaluating uncertainty against predictive error on the hold-out test set using the MU strategy, we observed that no molecules exhibited an uncertainty value below 0.4, indicating that the model was never very, or even moderately confident, in its predictions shown in Figure 12. Molecules with uncertainty values greater than 0.8 appeared only in the earliest iterations (0 and 1), with 55 instances (1.9%) in iteration 0 and a single instance in iteration 1. This reveals a key limitation of the method: it struggles to reinforce previously learned information, likely due to the constantly evolving feature landscape introduced by each new iteration of training data.

**Figure 12:**
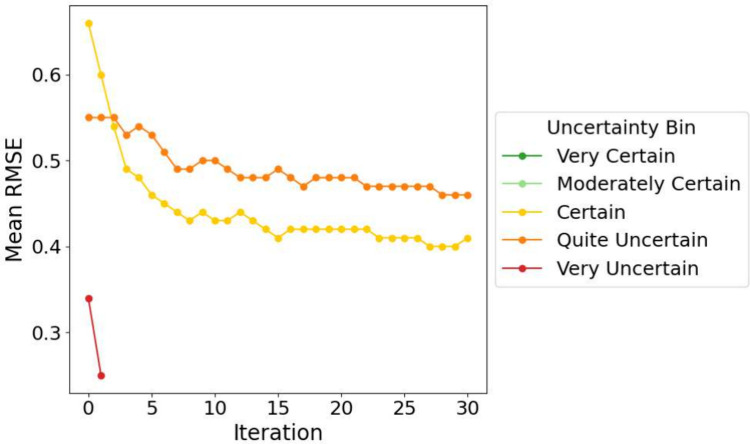
Root mean square error (RMSE) development across AL iterations using the MU strategy, with test set predictions binned by model uncertainty. Uncertainty bins are defined as follows: Very Certain (dark green): 0.0 < *x* ≤ 0.2; Moderately Certain (light green): 0.2 < *x* ≤ 0.4; Certain (orange): 0.4 < *x* ≤ 0.6; Quite Uncertain (yellow): 0.6 < *x* ≤ 0.8; Very Uncertain (red): *x* > 0.8

As a result, model predictions should not be treated at face value, and incorporating uncertainty directly into decision-making can provide a more cautious and informative approach.

When comparing the findings of this study to prior literature, both similarities and important differences emerge. Many previous studies report that random sampling is consistently less efficient than exploitative strategies, which contrasts with our results.^13,15,17,39^ This discrepancy likely arises from differences in the distributional relationship between training and test data. In most prior work, test compounds share similar or narrower feature distributions relative to the training set, making random sampling less informative. In our case, however, the PyMolGen-generated candidate molecules exhibit broader and more chemically diverse features compared to the ChEMBL-derived training set. Under these conditions, random sampling exposes the model to underrepresented regions of scaffold space, thereby improving its predictive performance within the chemical domain of interest. This indicates that random sampling is particularly important where the training set is derived from differentiated chemical matter, whether from external data, or from global or related project models. It also implies that random sampling is critical when there is an expectation that the features of the chemical space of interest is likely to drift; in reality, this is a possibility for all active discovery projects until high confidence in candidate identification is obtained.

Similar RMSE trends were reported by Khalak et al., who found that random sampling produced the greatest reduction in predictive error on compounds outside the training set, followed closely by uncertainty sampling, consistent with our results on the hold-out set.^40^ However, their findings diverge from ours with respect to hit-finding: despite improved prediction accuracy, both random and uncertainty-based strategies performed poorly in their equivalent of hit discovery, whereas in our study, uncertainty-based sampling achieved the highest hit rate.

Hybrid strategies in the literature are often associated with enhanced performance relative to single-pronged approaches. For example, Wang et al. and Gorantla et al. employed alternating exploration and exploitation schemes and reported improved model performance.^12,13^ This stands in contrast to our findings, where hybrid strategies did not outperform dedicated uncertainty-driven approaches.

A key distinction in our setup is the presence of distributional shift: the model is trained on a broad set of known BRD4 inhibitors from ChEMBL and applied to a chemically narrower scaffold space with limited experimental data. This shift increases the risk of overfitting for exploitative strategies and reduces their generalization to novel compounds. As previously discussed, this leads the model to overpredict for molecules with underrepresented features, conflating high uncertainty and high predicted potency.

To address this issue, some studies have incorporated uncertainty directly into the acquisition function. Reker et al. introduced a “conservative affinity estimate” by subtracting uncertainty from predicted affinities to penalize overconfident predictions.^11^ Similarly, Lonsdale et al. adjusted predicted values by their confidence estimates, either scaling them up or down, to balance exploitation and exploration.^41^ Such approaches could mitigate overprediction by replacing raw scores with weighted acquisition values that account for confidence, rather than relying solely on predicted potency or uncertainty.

Yin et al. highlighted systemic issues with ensemble methods, such as model ensembles and Monte Carlo Dropout, showing that they can be overconfident in unfamiliar regions of chemical space.^42^ However, while overconfidence may be problematic in static or single-shot learning contexts, it is less of a concern in iterative frameworks like ours. As the training set expands over successive DMTA cycles, the uncertainty landscape evolves, allowing the model to refine its confidence estimates and improve generalization. In this setting, uncertainty remains an effective signal for identifying regions of the chemical space where model predictions are less reliable.

## Conclusion

We developed an algorithm to simulate the pharmaceutical DMTA cycle in order to evaluate how different molecule selection strategies perform when applied to a narrow chemical domain derived from known inhibitors. Our aim was to investigate how ML models can effectively guide candidate selection in early stages of drug discovery such as ligand identification and optimization.

We found that actively querying molecules based on predictive uncertainty consistently yielded the greatest improvements in hit-finding performance across simulated iterations, identifying 37 hits within the top 50 predicted molecules (74%) for the final iteration. In contrast, exploitative strategies that prioritized top-scoring compounds failed to improve the model’s predictive ability and instead reinforced biases in the training data. Hybrid strategies provided limited benefit, often associating high docking scores with high uncertainty and yielding performance similar to purely greedy approaches.

These findings suggest that exploration of underrepresented regions in the learned feature space is critical for improving hit discovery, particularly when working with sparse data in narrowly defined chemical domains. Although high-uncertainty compounds may pose synthetic challenges, prioritizing those that are both accessible and informative offers a practical balance between diversity and feasibility.

We therefore propose selecting synthetically accessible molecules that rank highly in model uncertainty as an effective and pragmatic compromise. While this approach may not universally outperform all other strategies, as seen in the more favorable hits discovered in the RMP and RMPO strategies, it offers a strong balance between exploration and feasibility in earlier-stages of discovery campaigns.

It is important to note that our evaluation was limited to ligands for a single protein target and focused solely on predicted docking scores. Further work incorporating additional protein systems and other selection criteria deployed in real-world drug discovery pipelines such as ADMET properties will be necessary to assess the broader applicability of these findings. Furthermore, the present work investigates a restricted chemical domain, where the chemical scaffold is limited to only two R-group substitutions, simulating the lead identification or optimization stages. In other stages, where the data set contains a broader range of structures, other query strategies may prove to be more effective than the ones presented here, diversification of features within the training set becomes more likely.

## Supplementary Material



## Data Availability

The code used to simulate the DMTA cycle, along with the initial training data in CSV format is available at https://github.com/HuwJWilliams/SimDMTA.

## Notes

Additional experimental details, materials and methods, and results, including Figures S1–S18 and eqs 1–5 (PDF)
