# Supplementary material for: Query Matters: How Selection Strategies Influence Active Learning in Drug Discovery
Source: J Chem Inf Model. 2026 Feb 26;66(6):3288–301. doi: 10.1021/acs.jcim.5c02504 (PMC13014458; doi:10.1021/acs.jcim.5c02504)
Supplement: Supplementary file 1 [file ci5c02504_si_001.pdf]

Supporting Information:

Query Matters: How Selection Strategies Influence Active Learning in Drug Discovery

Huw J. Williams,† Stephen D. Pickett,‡ Andrew Baxter,‡ and David S. Palmer*,†

†*Department of Pure and Applied Chemistry, University of Strathclyde, 295 Cathedral Street, Glasgow, G11XL, United Kingdom.*

‡*GlaxoSmithKline R&D Pharmaceuticals, Stevenage, SG1 2NY, UK*

E-mail: david.palmer@strath.ac.uk

Contents of Supporting Information

Figure S1: Hit discovery rates for each individual resample of random-incorporating query strategies.

Equations 1 - 5: Pearson r, MSE, RMSE, SDEP, and Bias used for model evaluation.

Figure S2: Relationship between GNINA obtained docking scores and negative pIC_{50} values.

Figures S3 - S5: Distribution of docking scores for identified hits per strategy.

Figure S6: Average synthetic accessibility score of molecules added to the training set for MP, MPO, and MU.

Figures S7 - S8, S13 - S15: Evolution of top 10 important features (MP, MU, hybrid).

Figure S9: Average pairwise Tanimoto similarity of molecules added to the training set for MP, MPO, MU and R.

Figure S10: Hit discovery performance with a shuffled feature training set.

Figures S11 - S12: Model performance across single strategies with a shuffled feature training set.

Figures S16 - S18: Performance curves of hybrid strategies.

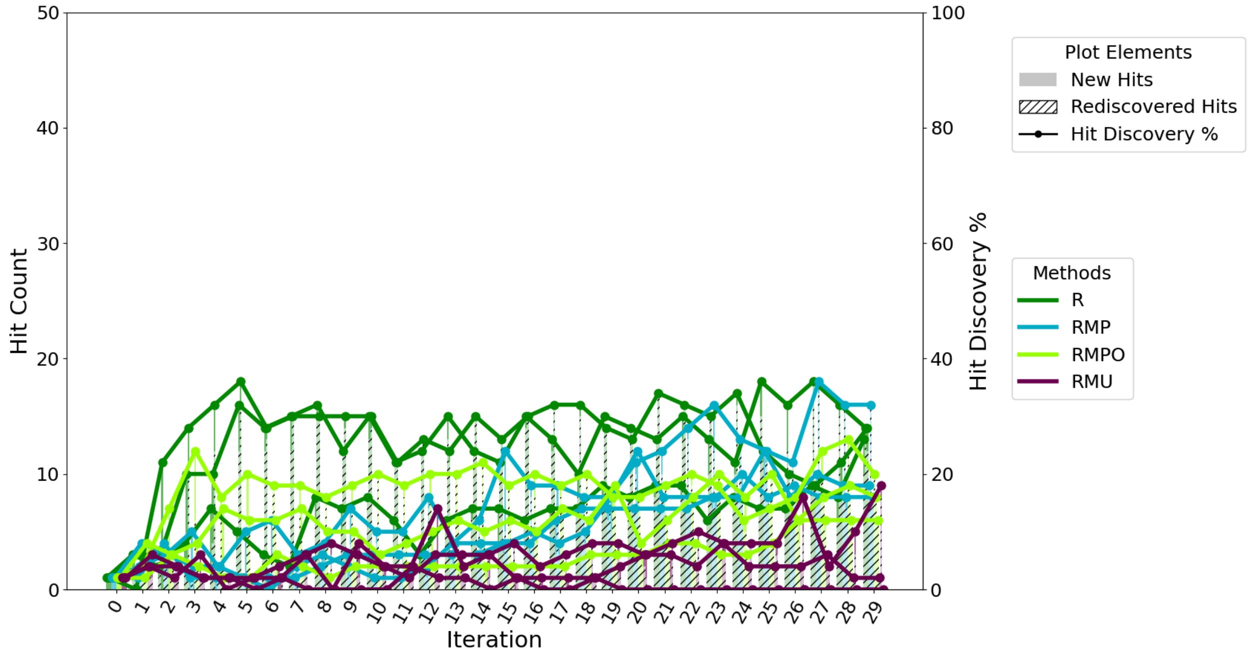

Figure S1: Hit discovery rates of each individual resample for the random-incorporating methods (R, RMP, RMPO and RMU)

$$PearsonR = \frac{\sum_{i=1}^{n}(y_i - \bar{y})(\hat{y}_i - \bar{\hat{y}})}{\sqrt{\sum_{i=1}^{n}(y_i - \bar{y})^2}\sqrt{\sum_{i=1}^{n}(\hat{y}_i - \bar{\hat{y}})^2}} \tag{1}$$

$$MSE = \frac{1}{n}\sum_{i=1}^{n}(y_i - \hat{y}_i)^2 \tag{2}$$

$$RMSE = \sqrt{MSE} \tag{3}$$

$$SDEP = \sqrt{\frac{1}{n}\sum_{i=1}^{n}(y_i - \hat{y}_i - Bias)^2} \tag{4}$$

$$Bias = \frac{1}{n}\sum_{i=1}^{n}(y_i - \hat{y}_i) \tag{5}$$

where n is the number of samples, y_i is the true value of the i-th sample, $\hat{y}_i$ is the predicted value for the i-th sample, and $\bar{y}$ is the mean of all true values.

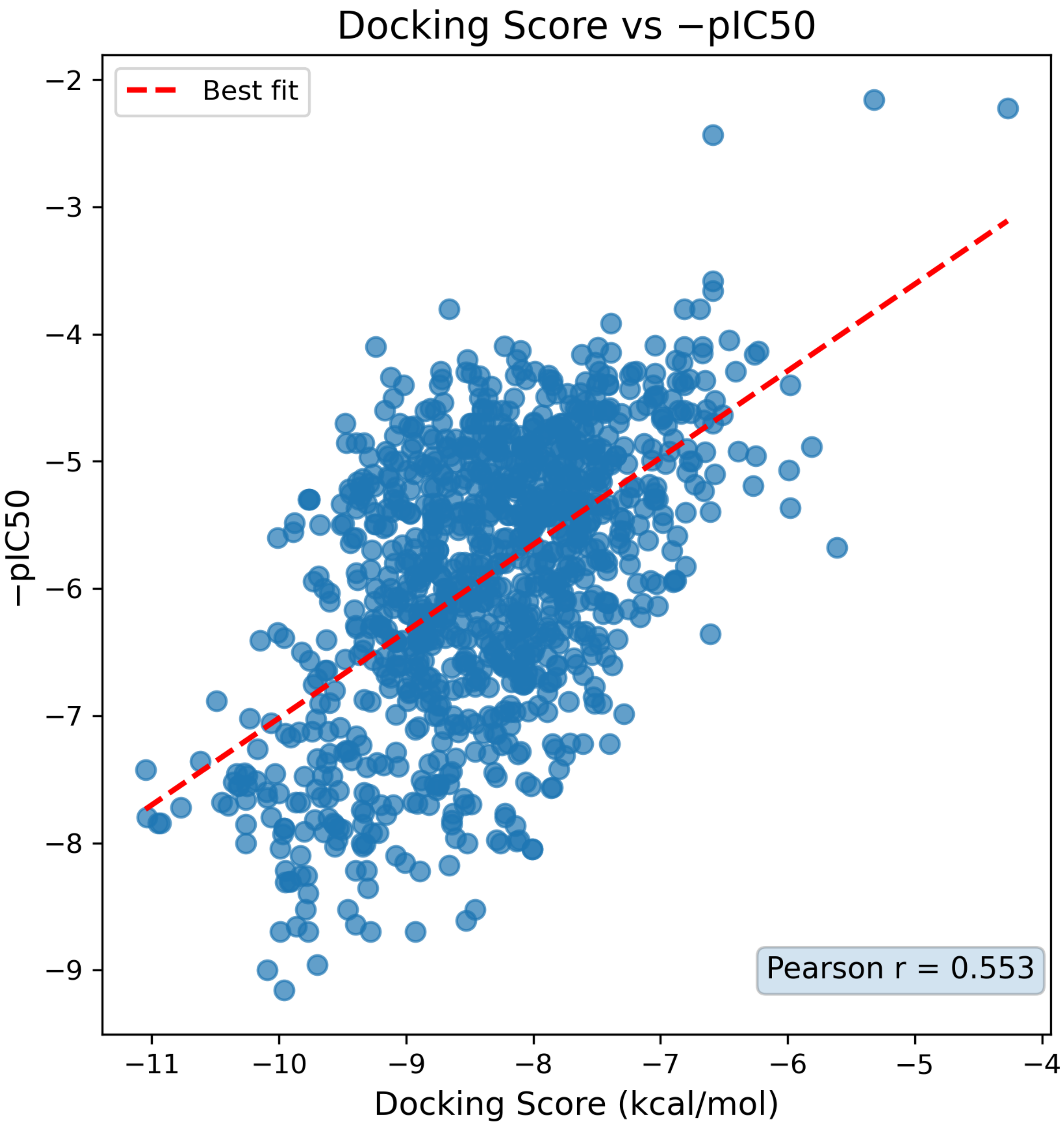

Figure S2: Scatter plot of docking scores (kcal/mol) versus negative pIC_{50} values for the molecules in the initial training set selected from ChEMBL.

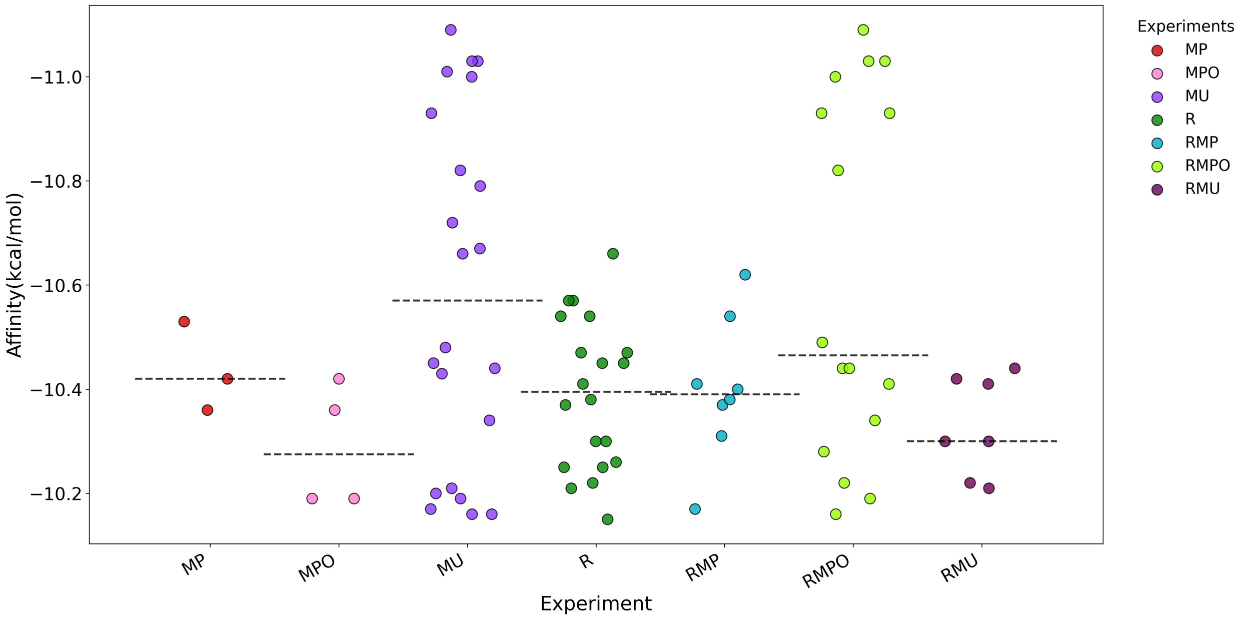

Figure S3: Distribution of true docking scores for discovered hits at iteration 10, grouped by selection strategy (MP, MPO, MU, R, RMP, RMPO and RMU). Hits are defined as compounds within the top 1 % of docking scores across the full evaluation set.

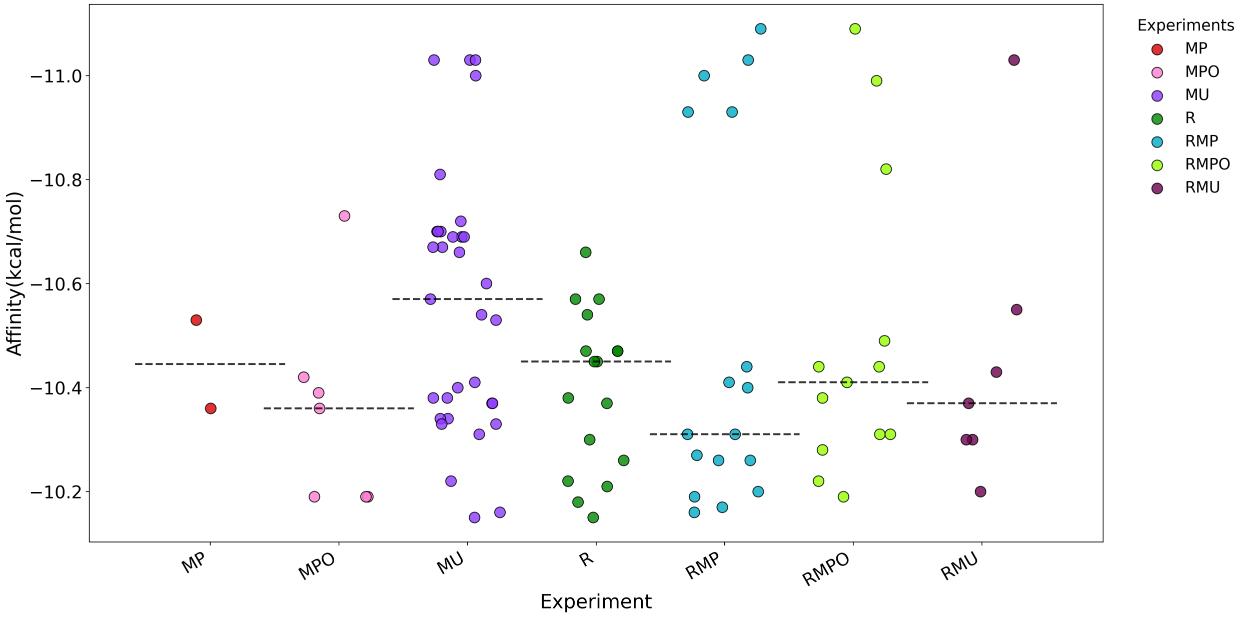

Figure S4: Distribution of true docking scores for discovered hits at iteration 20, grouped by selection strategy (MP, MPO, MU, R, RMP, RMPO and RMU). Hits are defined as compounds within the top 1 % of docking scores across the full evaluation set.

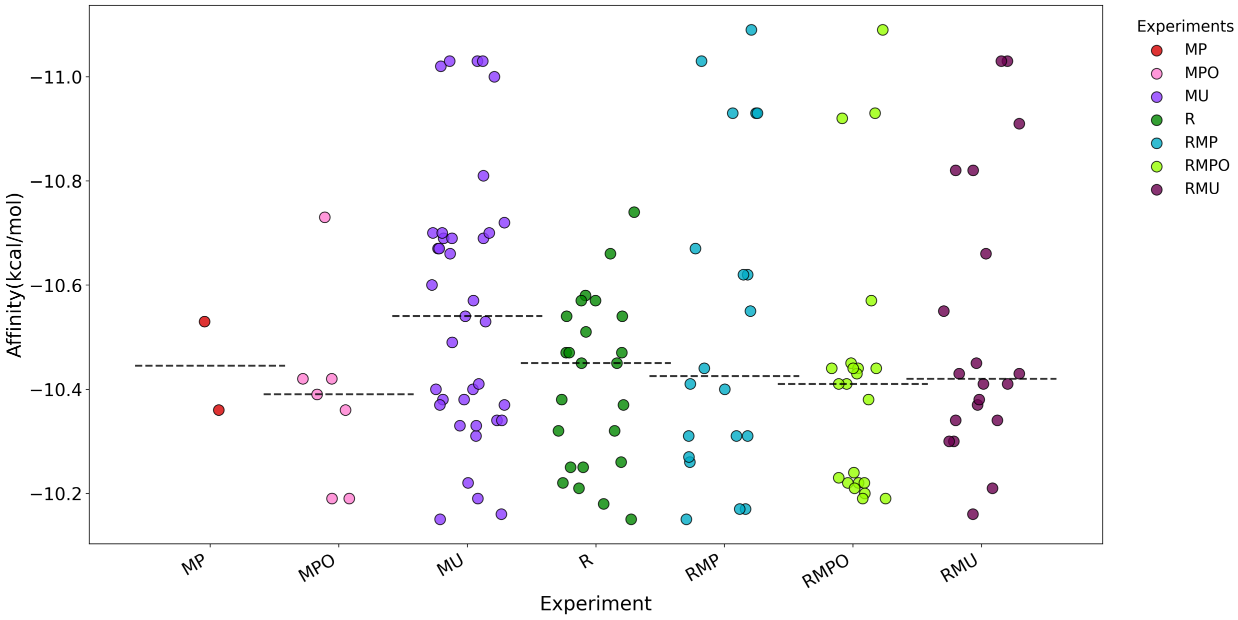

Figure S5: Distribution of true docking scores for discovered hits at iteration 30, grouped by selection strategy (MP, MPO, MU, R, RMP, RMPO and RMU). Hits are defined as compounds within the top 1 % of docking scores across the full evaluation set.

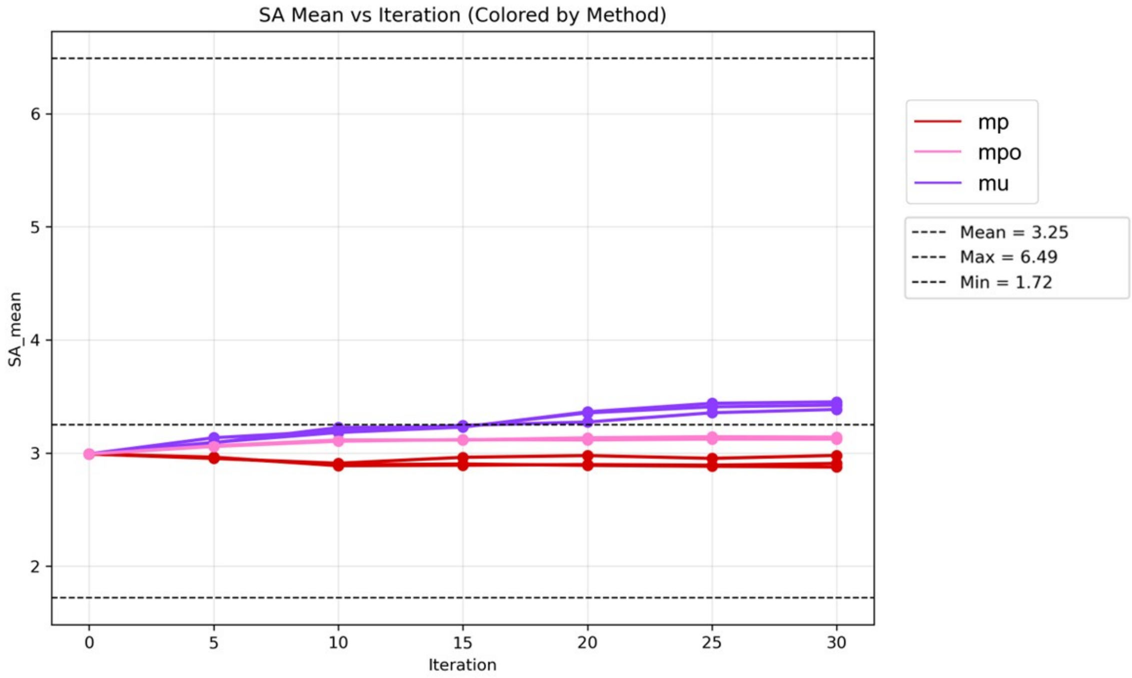

Figure S6: Average synthetic accessibility scores (SAScore) of selected molecules per iteration for each experiment. The minimum, mean, and maximum SAScores across all experiments were 1.72, 3.25, and 6.49, respectively. Query strategies shown: MP, MPO, and MU.

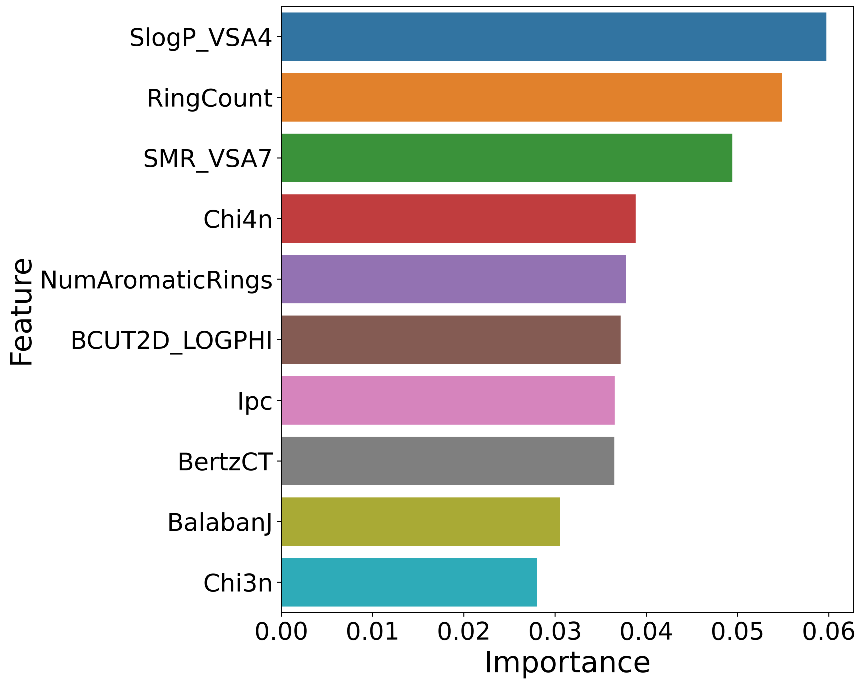

(a) Feature importance from the final iteration of the SimDMTA workflow using the MP query strategy, showing the top 10 features ranked by the random forest model.

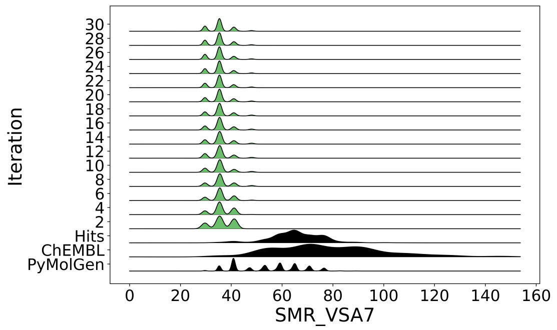

(b) SMR_VSA7

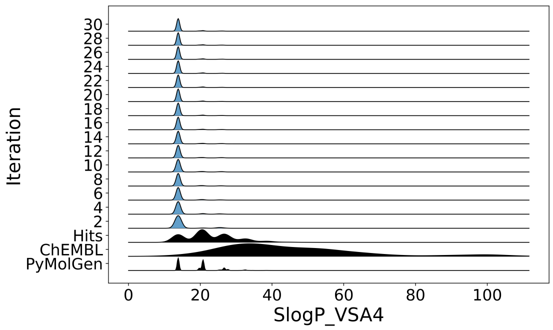

(c) SlogP_VSA4

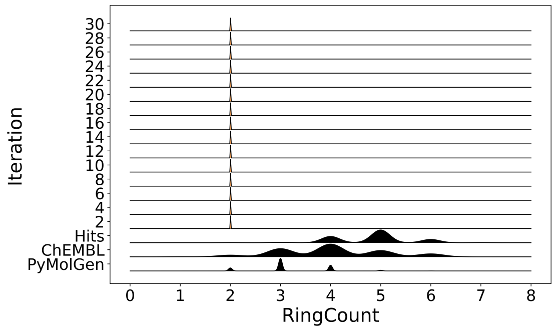

(d) RingCount

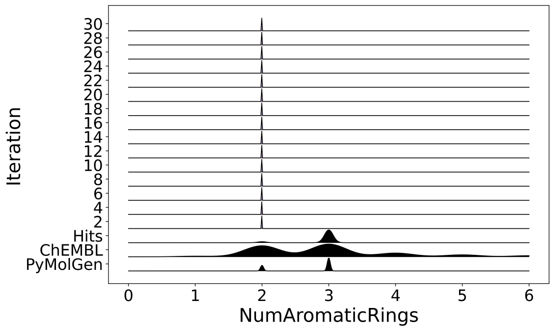

(e) NumAromaticRings

Figure S7: Feature distribution development across 30 iterations of the SimDMTA workflow using the MP query strategy, for the top 10 features ranked by RF importance in the final iteration.

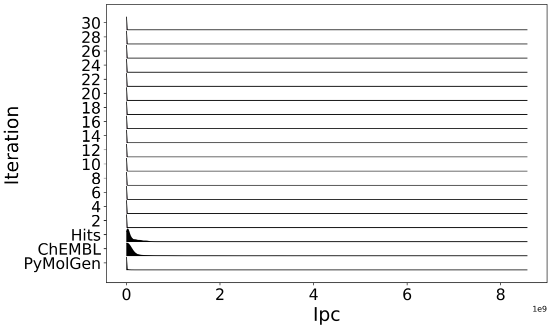

(f) Ipc

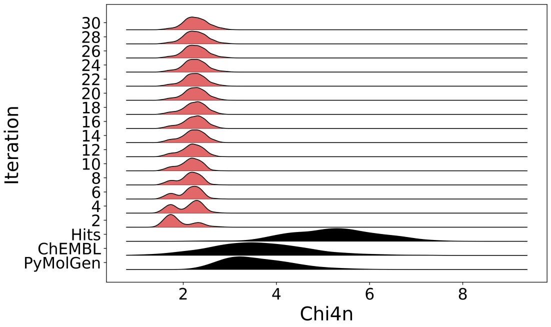

(g) Chi4n

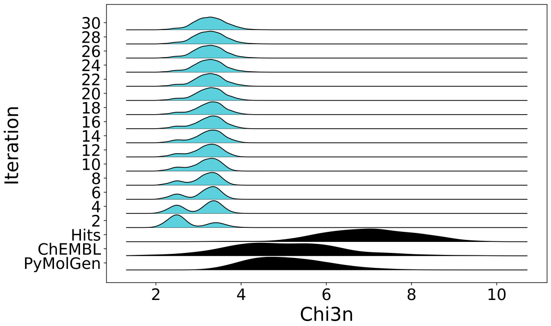

(h) Chi3n

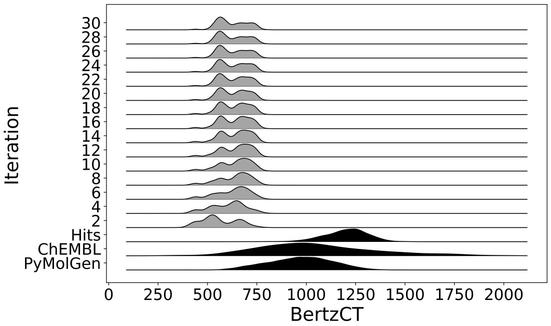

(i) BertzCT

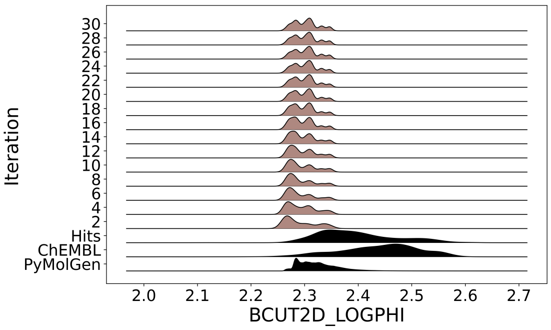

(j) BCUT2D_LOGPHI

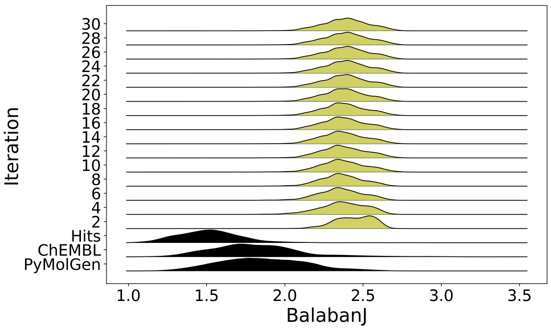

(k) Balabanj

Figure S7: (continued) Feature distribution development across 30 iterations of the SimDMTA workflow using the MP query strategy.

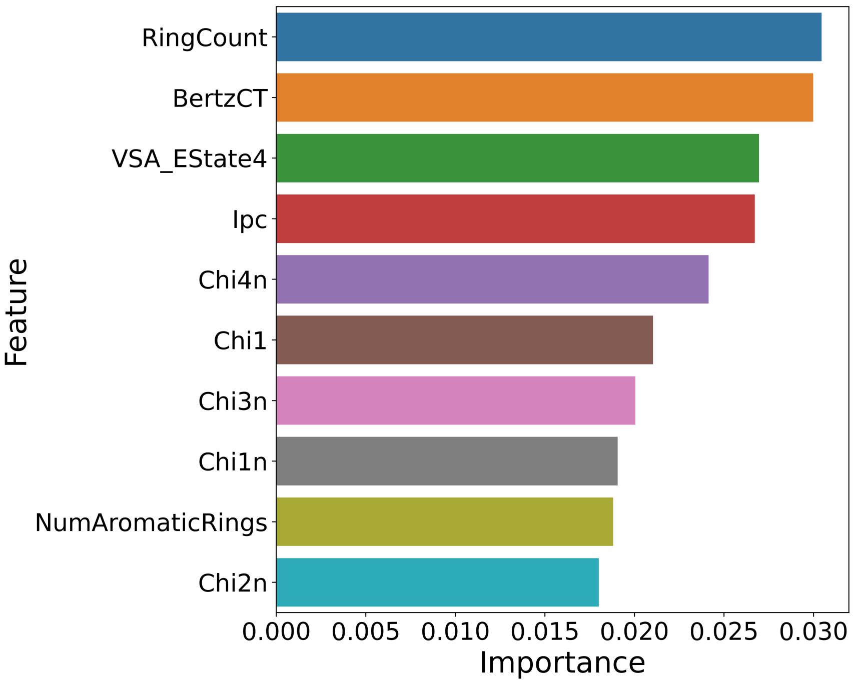

(a) Feature importance from the final iteration of the SimDMTA workflow using the MU query strategy, showing the top 10 features ranked by the random forest model.

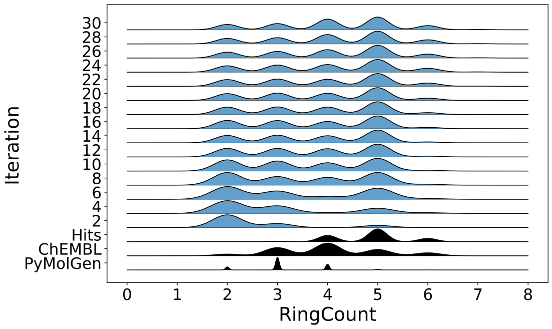

(b) SMR_VSA7

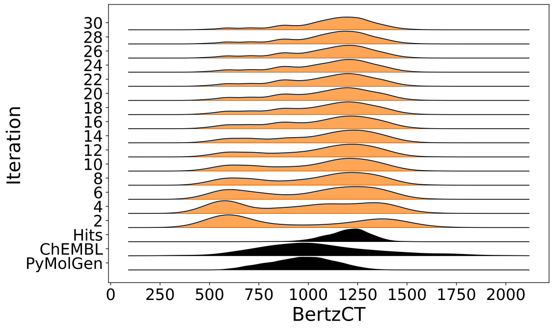

(c) SlogP_VSA4

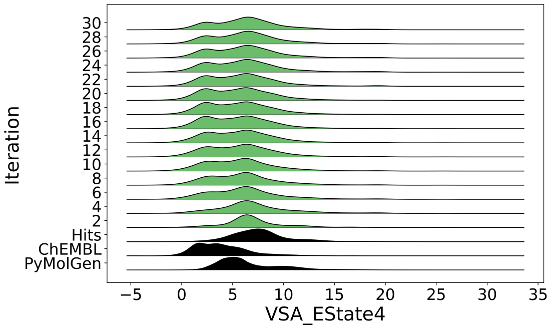

(d) VSA_EState4

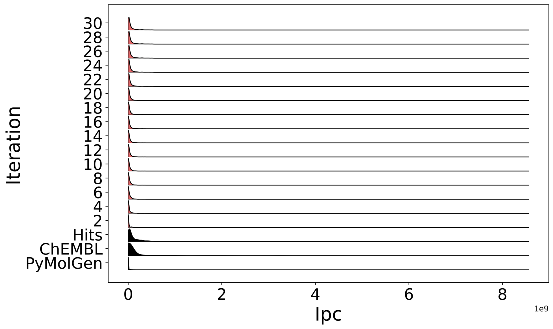

(e) Ipc

Figure S8: Feature distribution development across 30 iterations of the SimDMTA workflow using the MU query strategy, for the top 10 features ranked by RF importance in the final iteration.

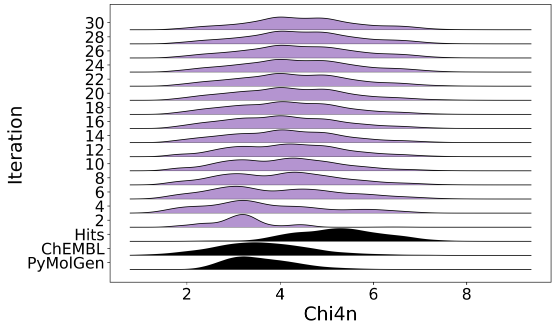

(f) Chi4n

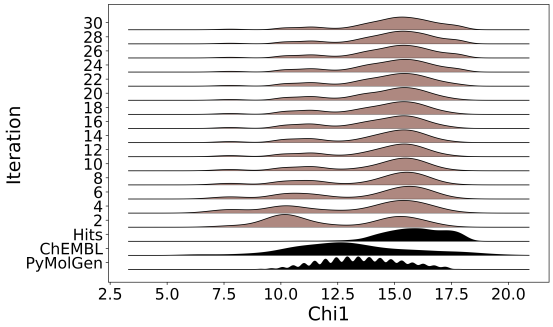

(g) Chi1

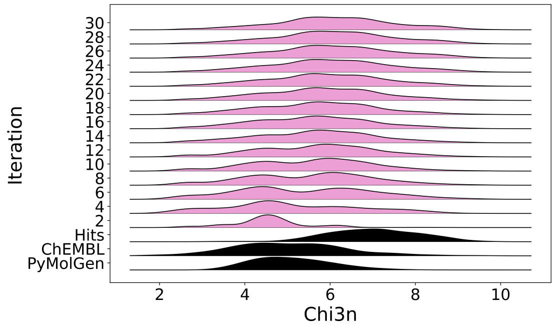

(h) Chi3n

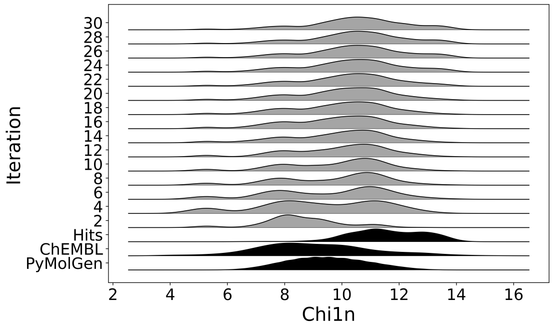

(i) Chi1n

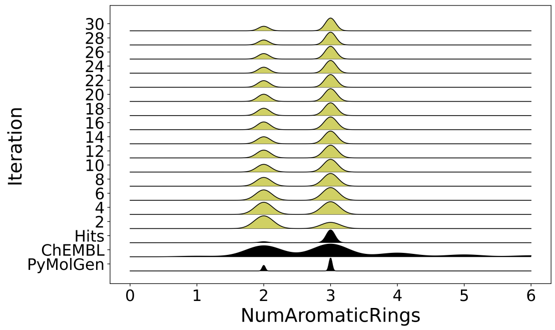

(j) NumAromaticRings

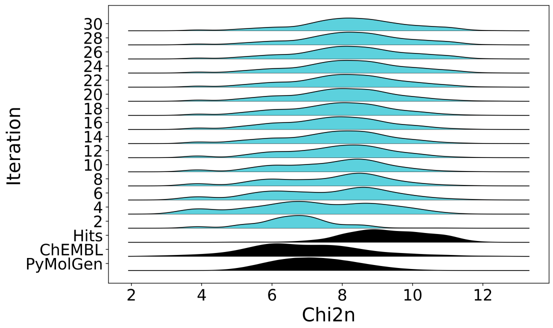

(k) Chi2n

Figure S8: (continued) Feature distribution development across 30 iterations of the SimDMTA workflow using the MU query strategy.

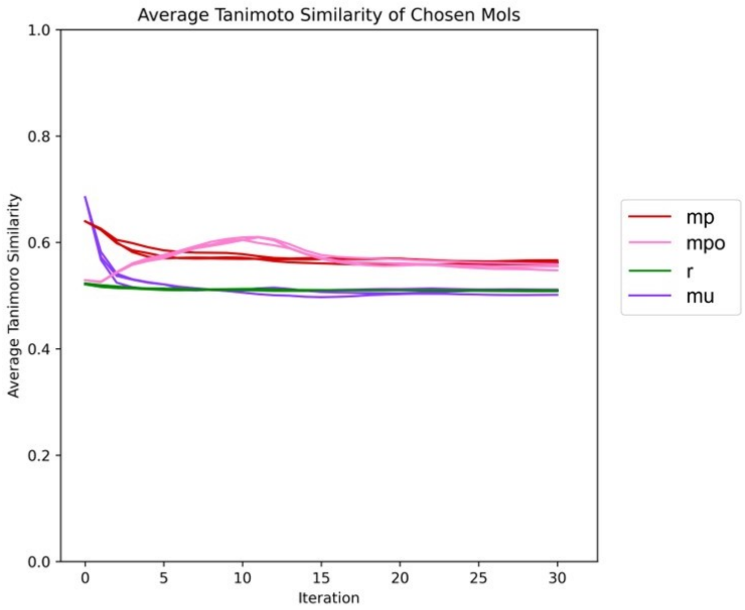

Figure S9: The average Tanimoto similarity calculated between all pairs of molecules within the set of molecules selected at each iteration. Comparison of query strategies: MP, MPO, R, and MU.

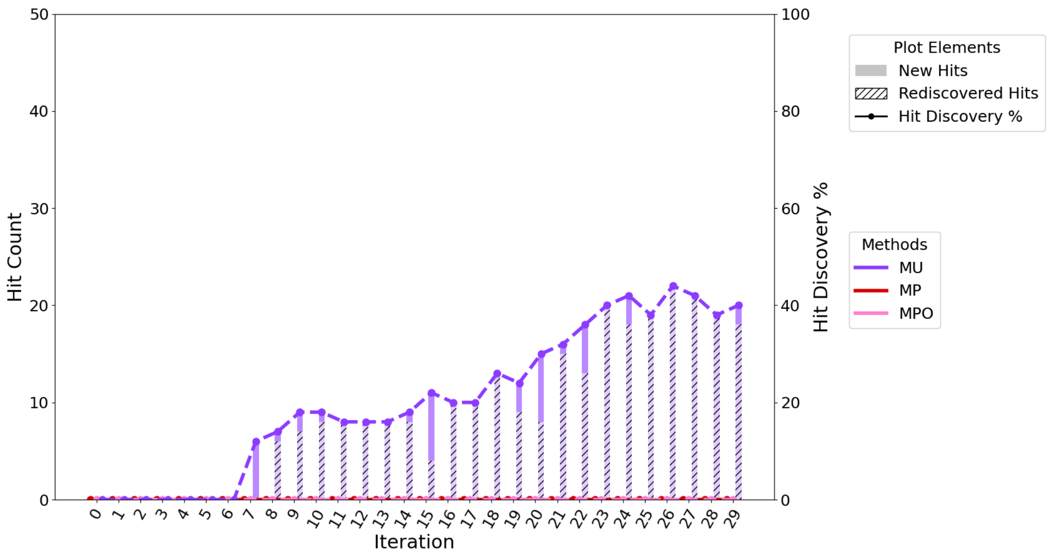

(a) Top-ranked selection from a 'shuffled start'

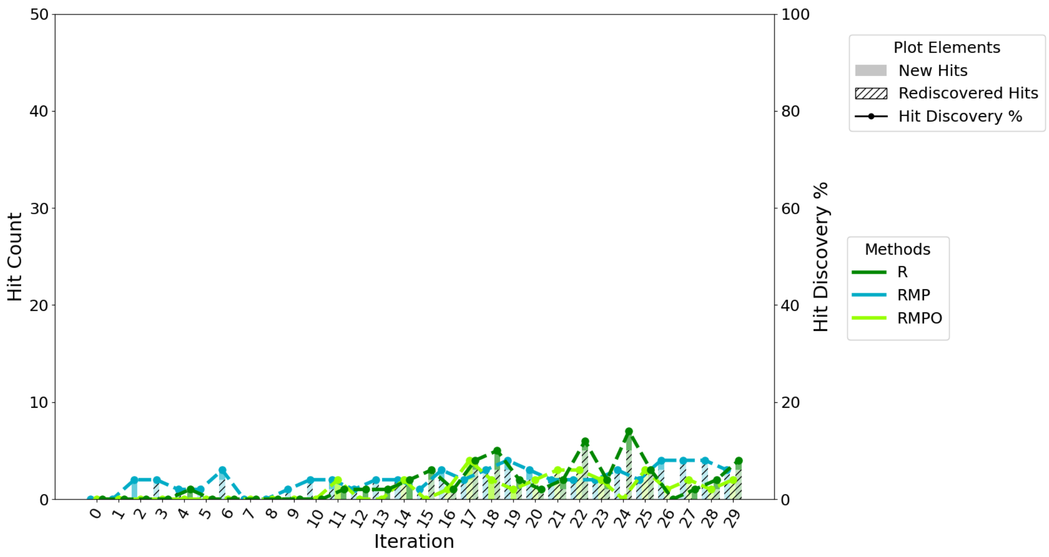

(b) Randomized top-10% selection from a 'shuffled start'

Figure S10: Hit discovery performance of acquisition strategies across iterations with iteration 0 using uncorrelated (shuffled) features. Hits are defined as the top 1% of all docked compounds based on actual docking scores. (a) Compounds selected strictly by top acquisition scores (MP, MPO, MU). (b) Compounds selected either purely at random or randomly within the top 10% of each acquisition score ranking.

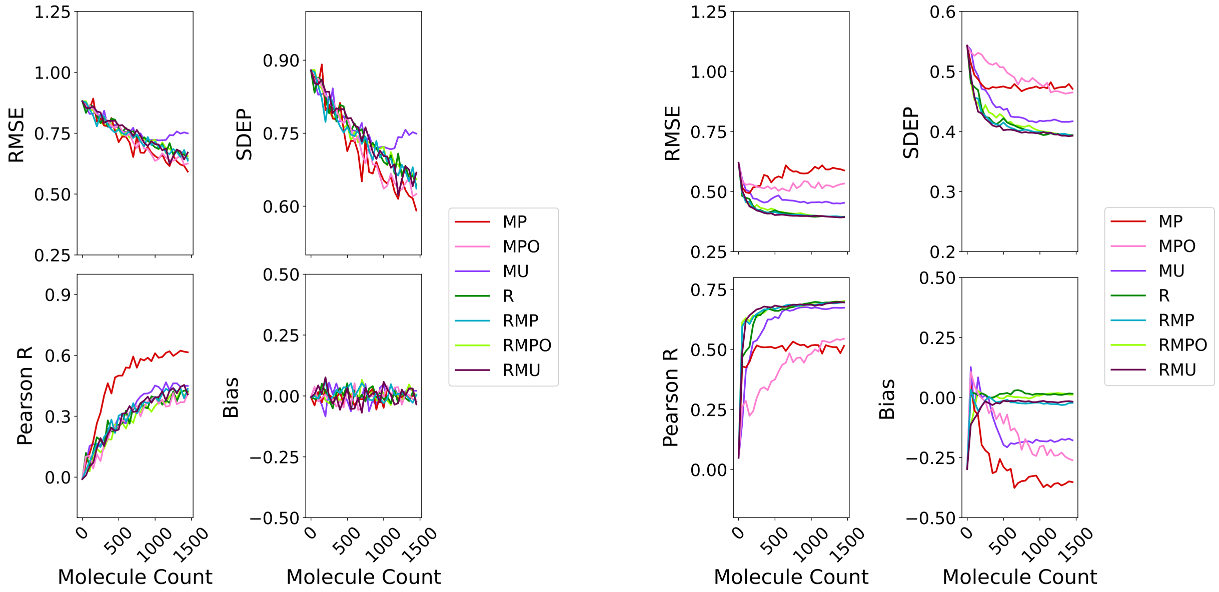

(a) Internal (b) Hold Out

Figure S11: Average predictive performance across all query strategies (batch size = 50) with a feature-randomized starting point, showing internal performance during retraining (a), predictive performance on entire hold out test set (b). The x-axis shows the cumulative number of molecules added to the training data during active learning.

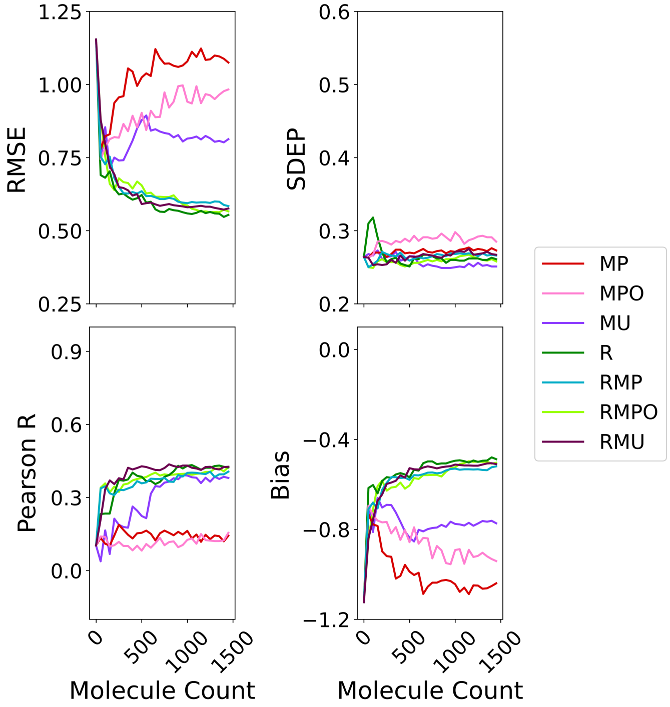

Figure S12: Average predictive performance across all query strategies (batch size = 50) with a feature-randomized starting point, evaluated on the top 500 molecules with the highest true docking scores from the hold-out test set. The x-axis shows the cumulative number of molecules added to the training data during active learning.

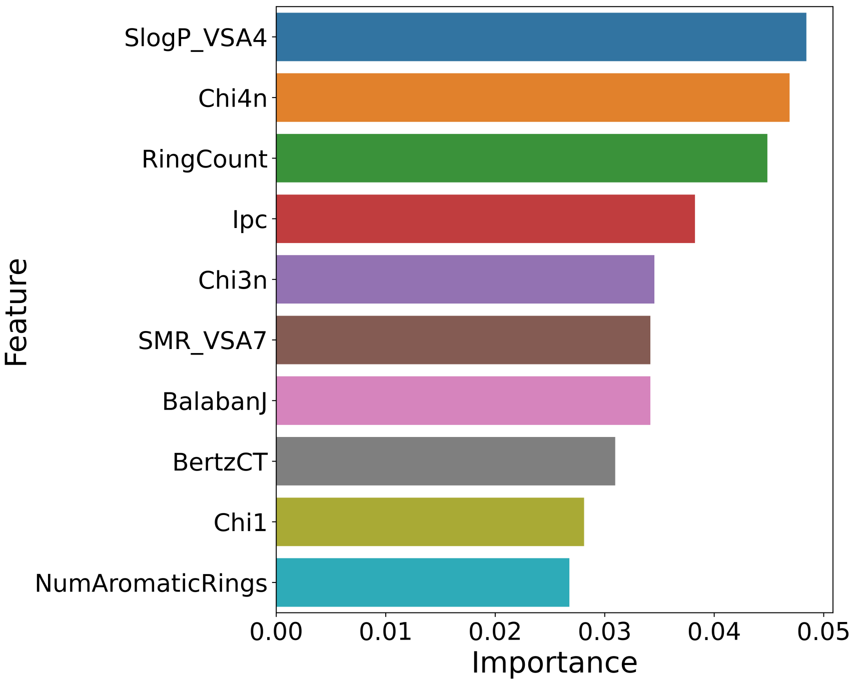

(a) Feature importance from the final iteration of the SimDMTA workflow using the MP:MU (2:8) hybrid query strategy, showing the top 10 features ranked by the random forest model.

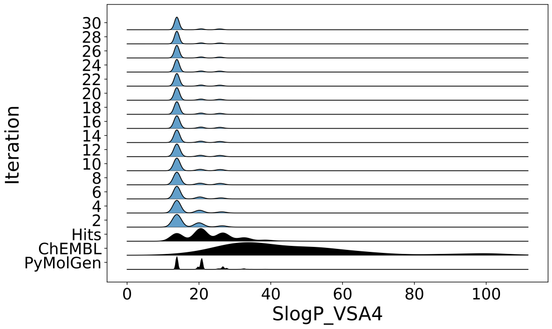

(b) SlogP_VSA4

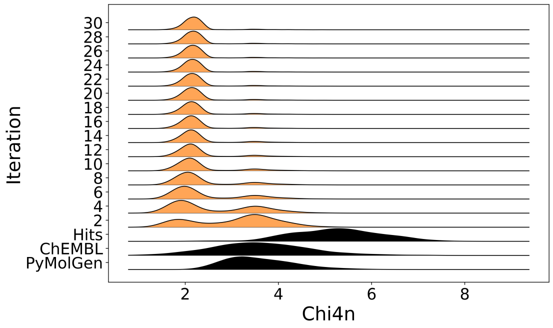

(c) Chi4n

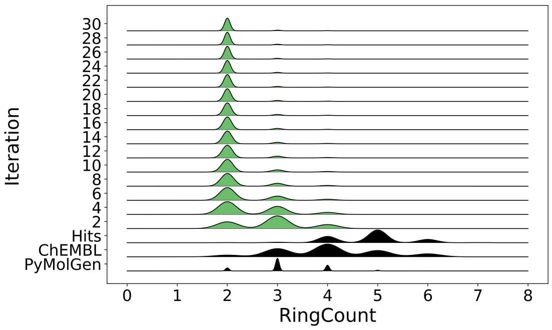

(d) RingCount

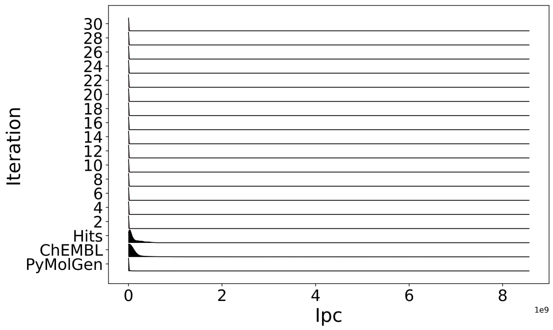

(e) Ipc

Figure S13: Feature distribution development across 30 iterations of the SimDMTA workflow using the MP:MU (2:8) hybrid query strategy, for the top 10 features ranked by RF importance in the final iteration.

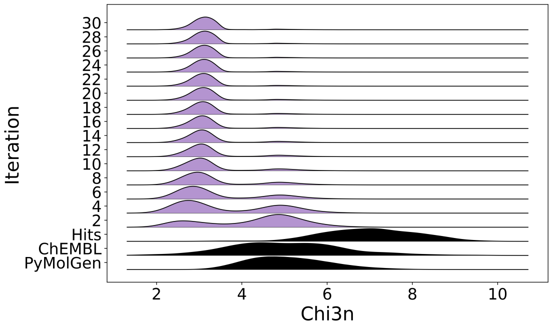

(f) Chi3n

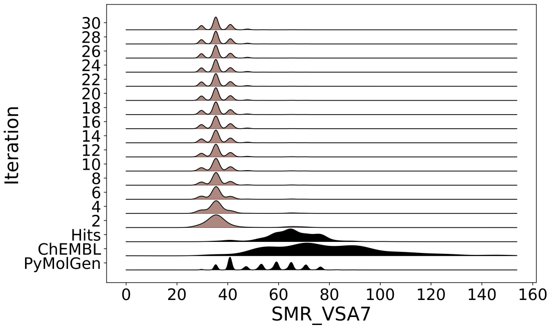

(g) SMR_VSA7

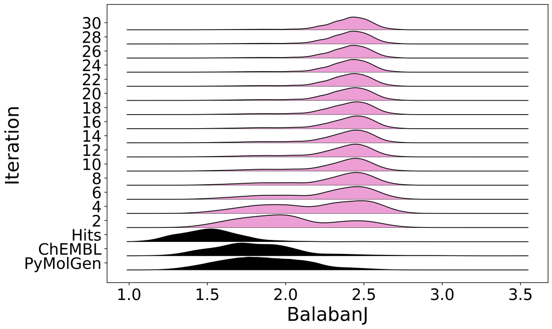

(h) Balabanj

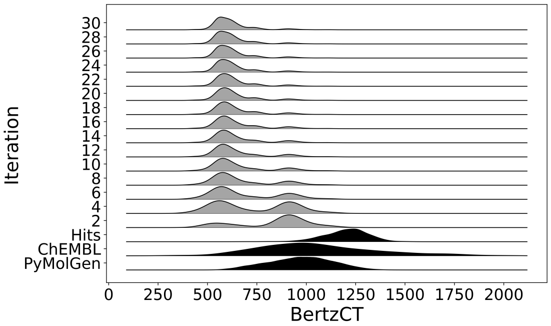

(i) BertzCT

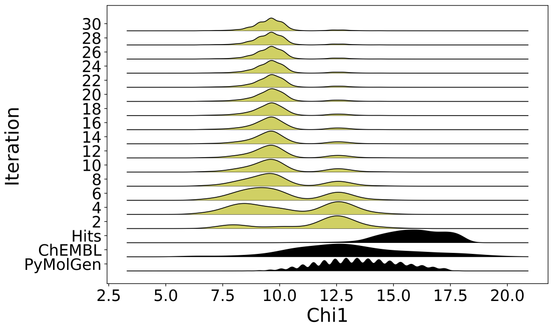

(j) Chi1

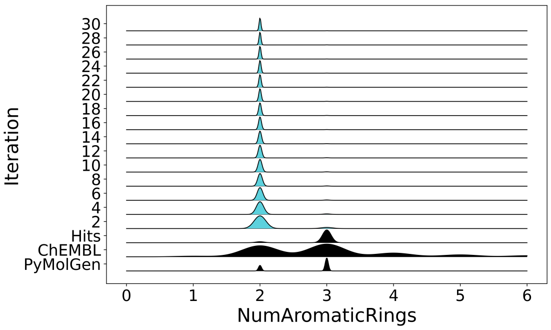

(k) NumAromaticRings

Figure S13: (continued) Feature distribution development across 30 iterations of the SimDMTA workflow using the MP:MU (2:8) hybrid query strategy.

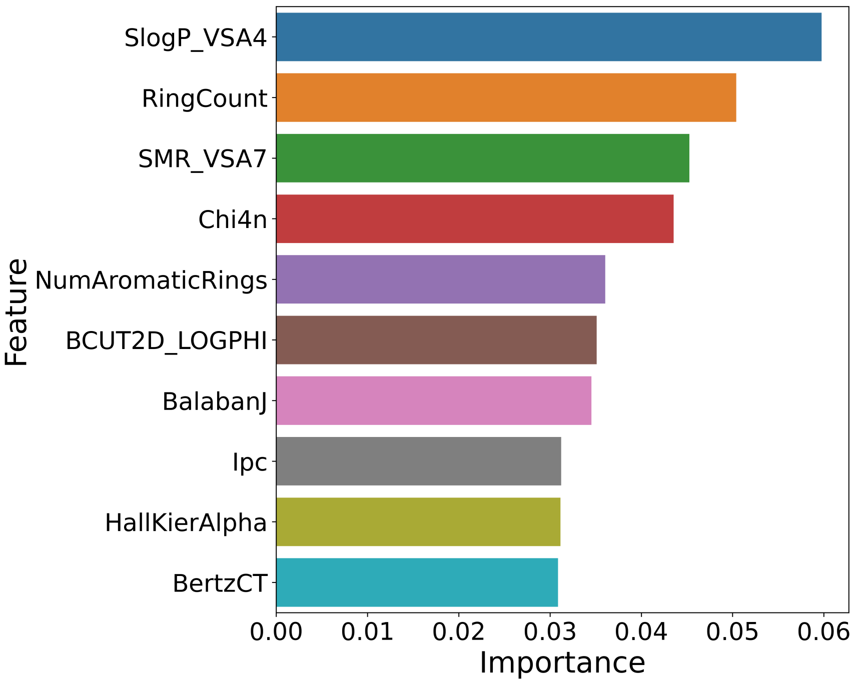

(a) Feature importance from the final iteration of the SimDMTA workflow using the MP:MU (5:5) hybrid query strategy, showing the top 10 features ranked by the random forest model.

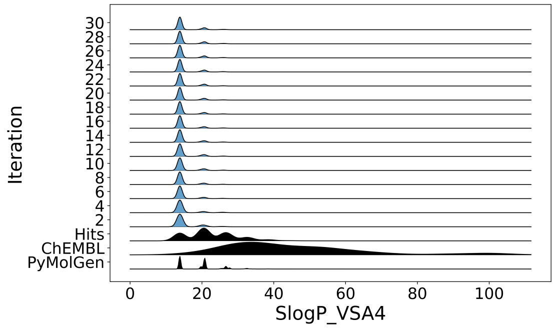

(b) SlogP_VSA4

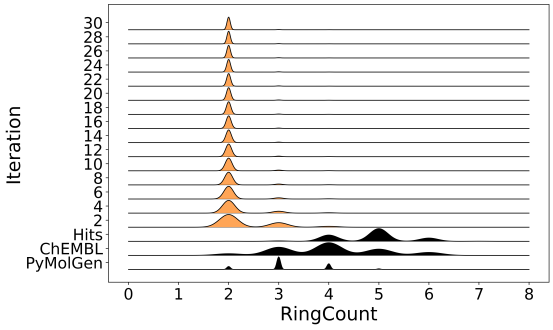

(c) RingCount

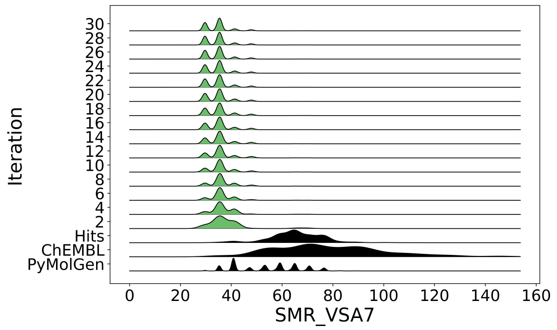

(d) SMR_VSA7

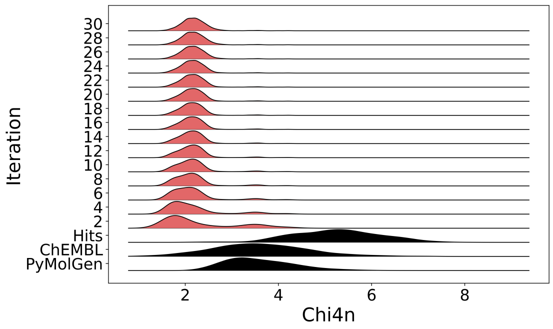

(e) Chi4n

Figure S14: Feature distribution development across 30 iterations of the SimDMTA workflow using the MP:MU (5:5) hybrid query strategy, for the top 10 features ranked by RF importance in the final iteration.

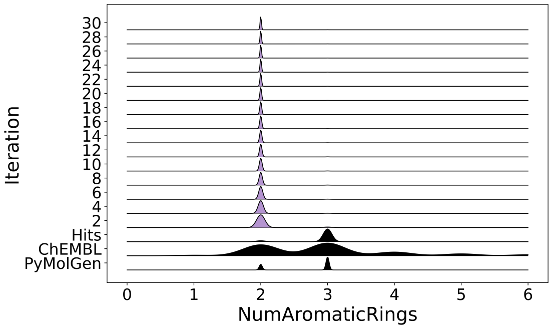

(f) NumAromaticRings

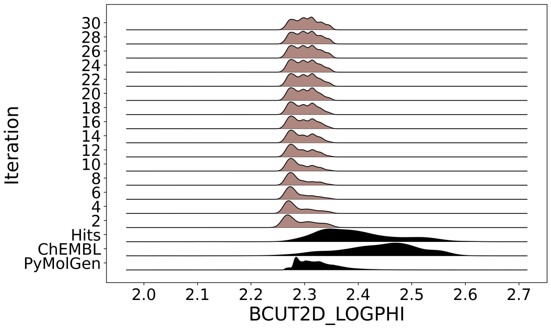

(g) BCUT2D_LOGPHI

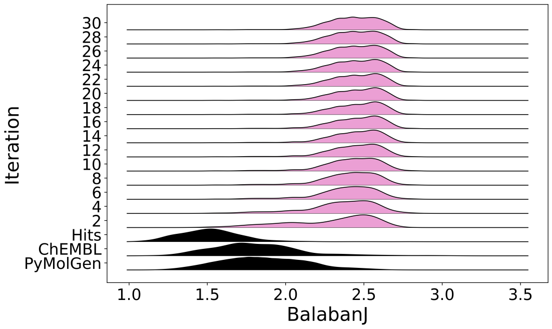

(h) Balabanj

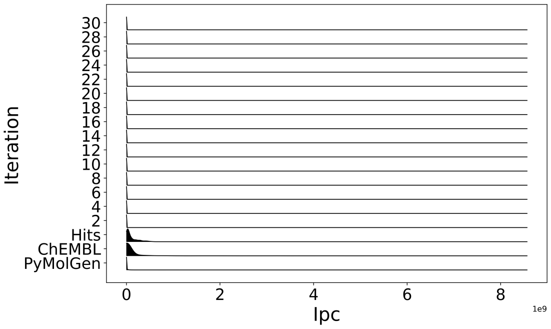

(i) Ipc

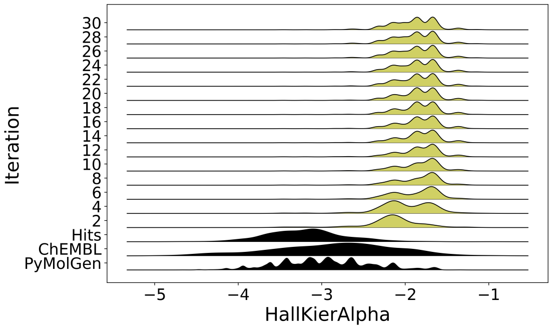

(j) HallKierAlpha

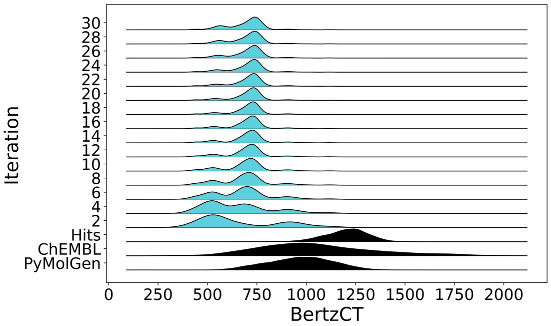

(k) BertzCT

Figure S14: (continued) Feature distribution development across 30 iterations of the SimDMTA workflow using the MP:MU (5:5) hybrid query strategy.

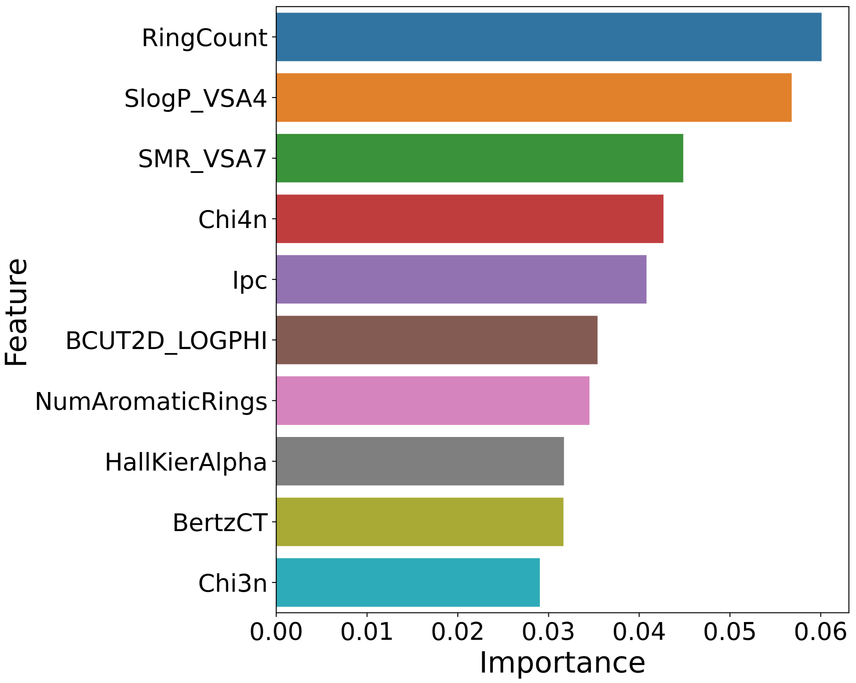

(a) Feature importance from the final iteration of the SimDMTA workflow using the MP:MU (8:2) hybrid query strategy, showing the top 10 features ranked by the random forest model.

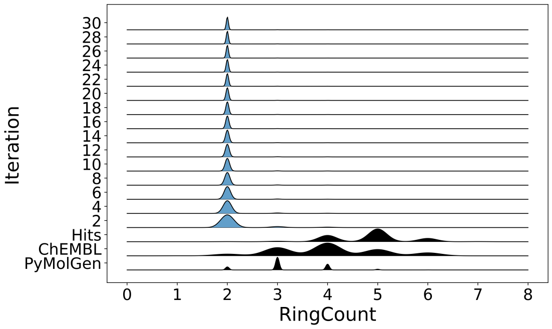

(b) RingCount

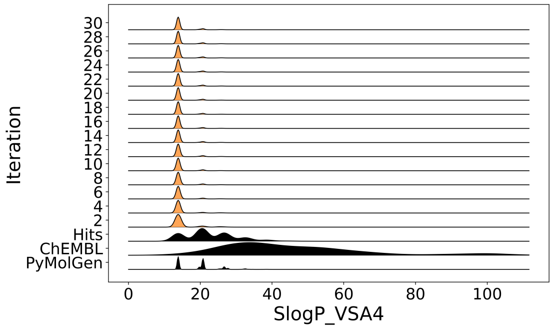

(c) SlogP_VSA4

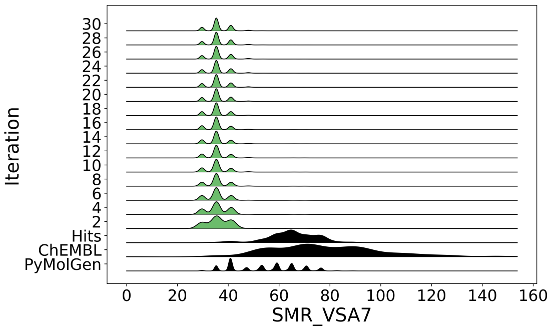

(d) SMR_VSA7

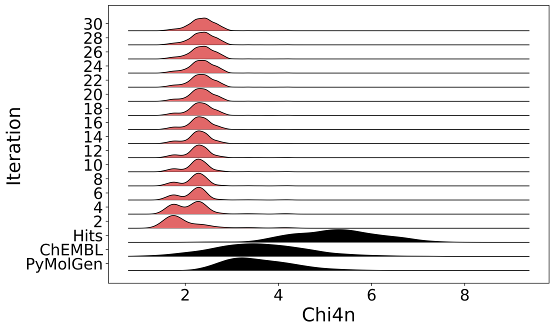

(e) Chi4n

Figure S15: Feature distribution development across 30 iterations of the SimDMTA workflow using the MP:MU (8:2) hybrid query strategy, for the top 10 features ranked by RF importance in the final iteration.

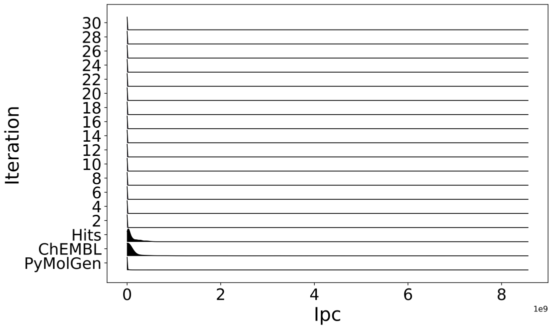

(f) Ipc

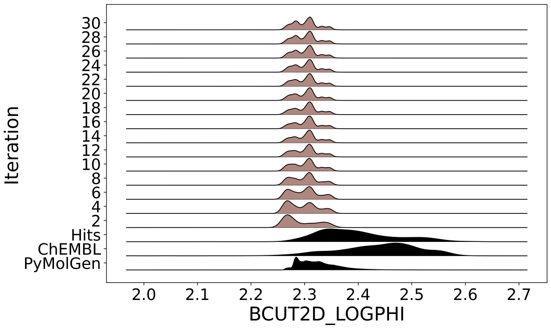

(g) BCUT2D_LOGPHI

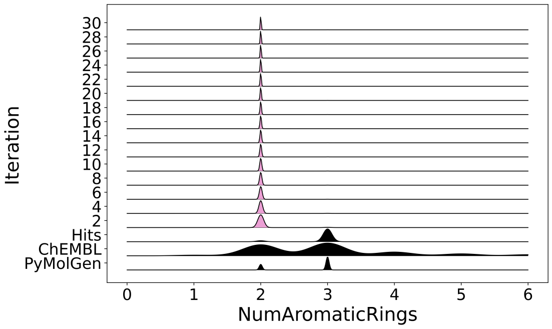

(h) NumAromaticRings

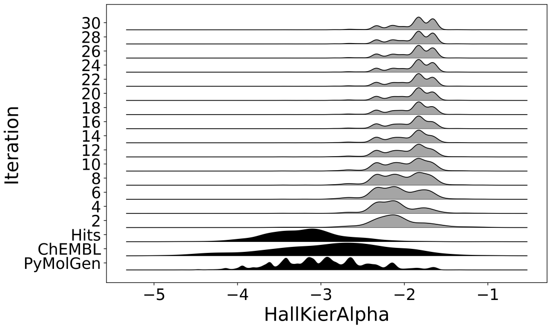

(i) HallKierAlpha

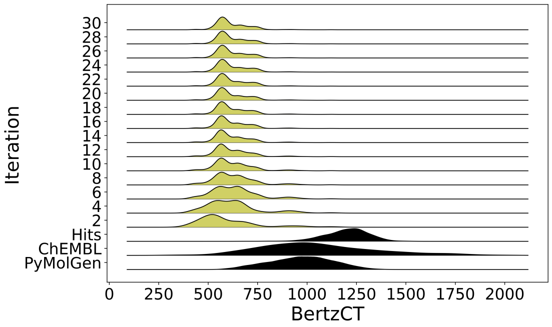

(j) BertzCT

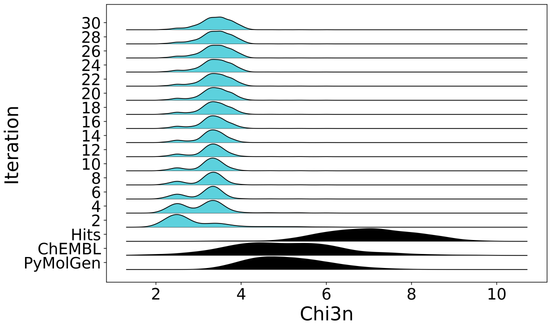

(k) Chi3n

Figure S15: (continued) Feature distribution development across 30 iterations of the SimDMTA workflow using the MP:MU (8:2) hybrid query strategy.

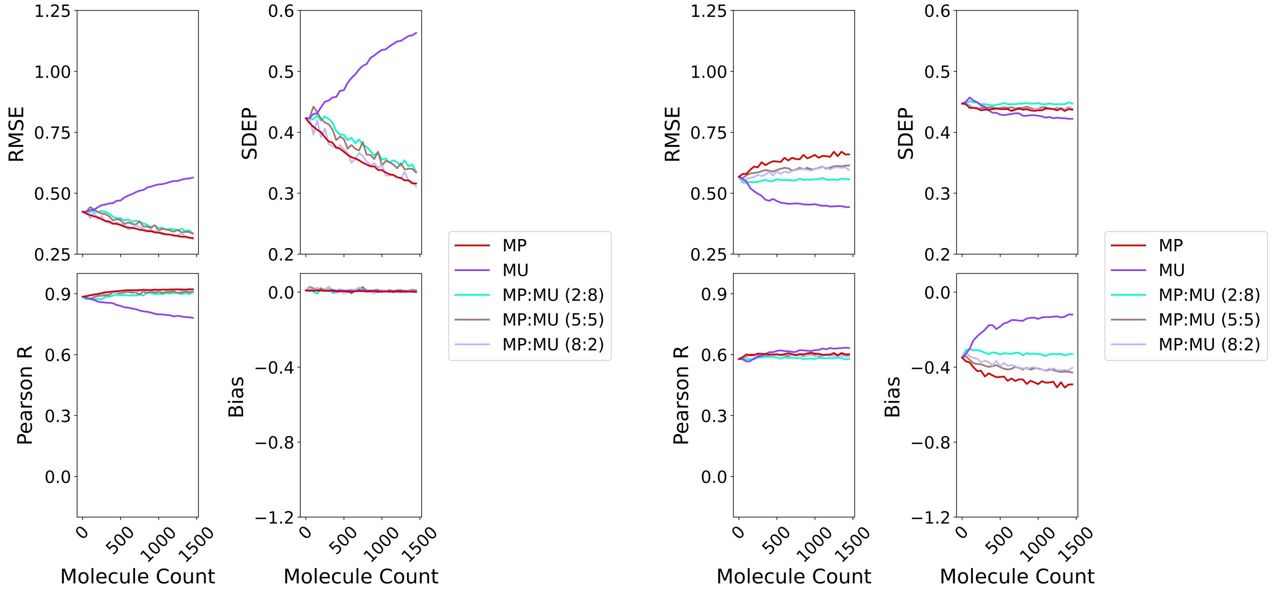

(a) Internal (b) Hold Out

Figure S16: Average predictive performance of the MP & MU hybrid strategy (batch size = 50) across three selection ratios (2:8, 5:5, and 8:2), showing (a) internal performance during retraining and (b) predictive performance on the full hold-out test set. The x-axis shows the cumulative number of molecules added to the training data during active learning.

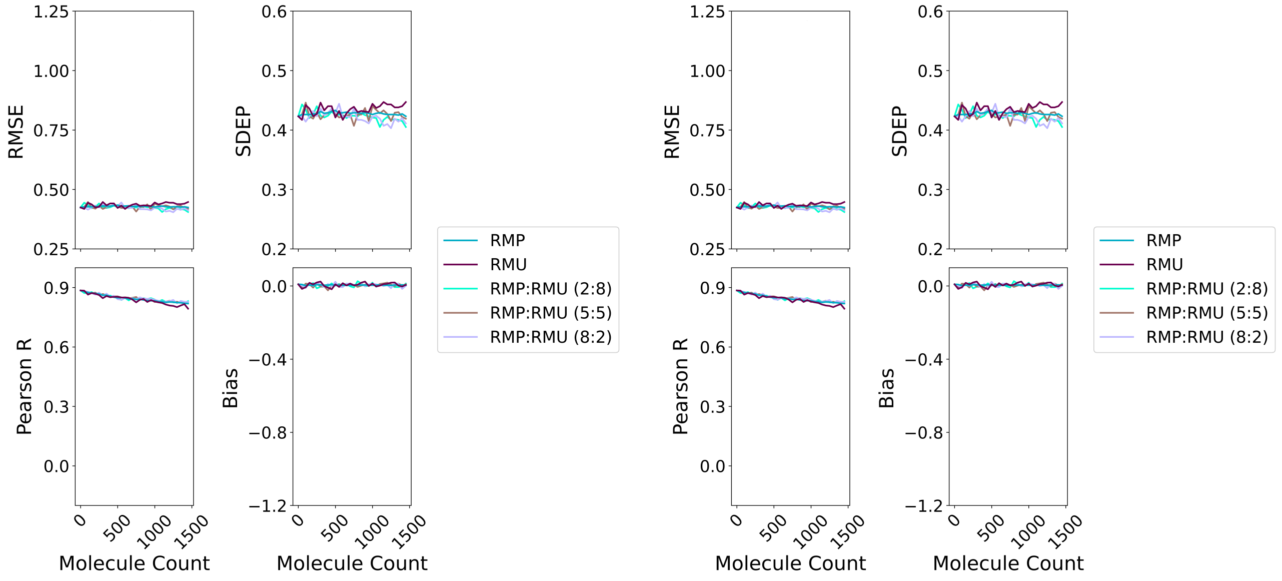

(a) Internal

(b) Hold Out

Figure S17: Average predictive performance of the RMP & RMU hybrid strategy (batch size = 50) across three selection ratios (2:8, 5:5, and 8:2), showing (a) internal performance during retraining and (b) predictive performance on the full hold-out test set. The x-axis shows the cumulative number of molecules added to the training data during active learning.

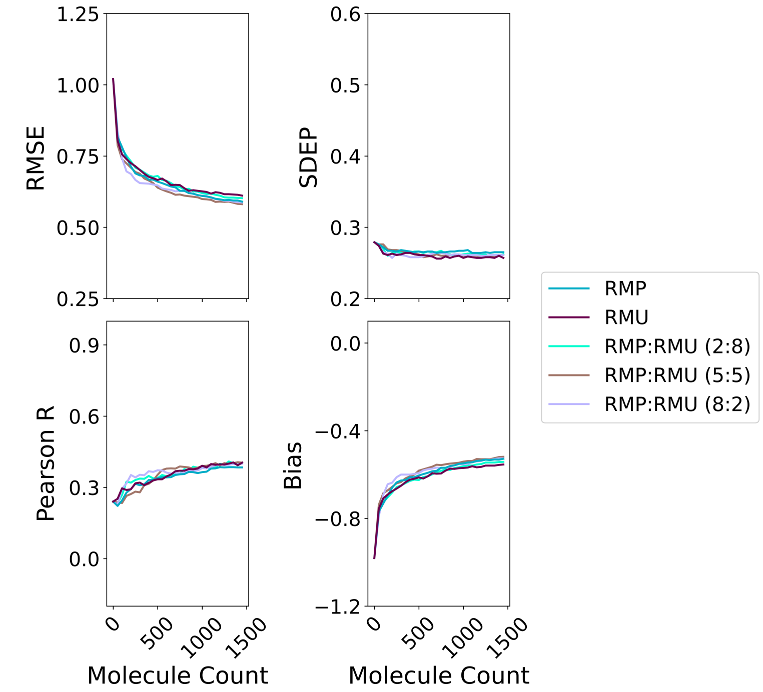

Figure S18: Average predictive performance across all the RMP & RMU query strategies (batch size = 50), evaluated on the top 500 molecules with the highest true docking scores from the hold-out test set. The x-axis shows the cumulative number of molecules added to the training data during active learning.
